# Regulation of folate and methionine metabolism by multisite phosphorylation of human methylenetetrahydrofolate reductase

**DOI:** 10.1038/s41598-019-40950-7

**Published:** 2019-03-12

**Authors:** Yuxiang Zheng, Shivan Ramsamooj, Qian Li, Jared L. Johnson, Tomer M. Yaron, Klaus Sharra, Lewis C. Cantley

**Affiliations:** 1 Meyer Cancer Center, Weill Cornell Medicine, New York, USA; 2 Institute for Computational Biomedicine, Department of Physiology and Biophysics, Weill Cornell Medicine, New York, USA

## Abstract

Methylenetetrahydrofolate reductase (MTHFR) catalyzes the irreversible conversion of 5,10-methylene-tetrahydrofolate (THF) to 5-methyl-THF, thereby committing one-carbon units to the methionine cycle. While MTHFR has long been known to be allosterically inhibited by S-adenosylmethionine (SAM), only relatively recently has N-terminal multisite phosphorylation been shown to provide an additional layer of regulation. *In vitro*, the multiply phosphorylated form of MTHFR is more sensitive to allosteric inhibition by SAM. Here we sought to investigate the kinases responsible for MTHFR multisite phosphorylation and the physiological function of MTHFR phosphorylation in cells. We identified DYRK1A/2 and GSK3A/B among the kinases that phosphorylate MTHFR. In addition, we found that MTHFR phosphorylation is maintained by adequate cellular SAM levels, which are sensed through the C-terminal SAM binding domain of MTHFR. To understand the function of MTHFR phosphorylation in cells, we generated MTHFR CRISPR knockin mutant lines that effectively abolished MTHFR phosphorylation and compared them with the parental cell lines. Whereas the parental cell lines showed increased 5-methyl-THF production in response to homocysteine treatment, the knockin cell lines had high basal levels of 5-methyl-THF and did not respond to homocysteine treatment. Overall, our results suggest that MTHFR multisite phosphorylation coordinates with SAM binding to inhibit MTHFR activity in cells.

## Introduction

**Figure 1:**
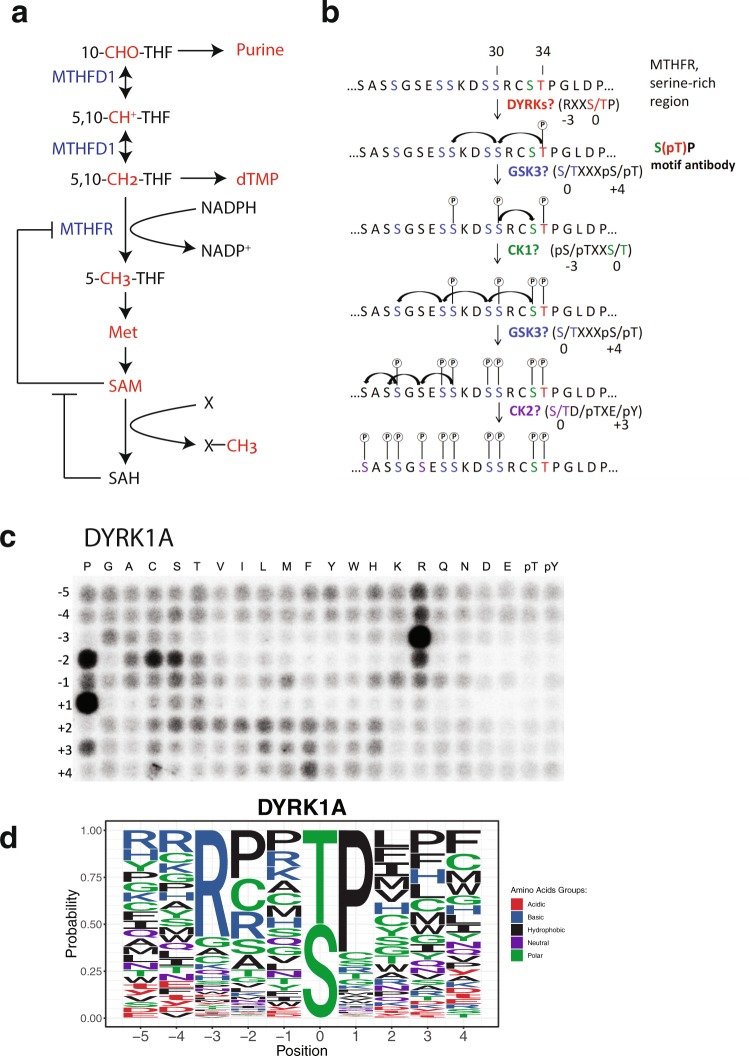
Regulation of MTHFR by allosteric inhibition and by phosphorylation. (**a**) MTHFR commits one-carbon units to methionine regeneration, and is allosterically inhibited by SAM and disinhibited by SAH. (**b**) Proposed scheme for the N-terminal multisite phosphorylation of MTHFR. Simplified substrate consensus motifs are shown here in parenthesis, whereas detailed logos are provided in Fig. S1. (**c**) Peptide library result for DYRK1A. Recombinant DYRK1A was distributed across a 384-well plate, mixed with the peptide substrate library, Kinase Assay Buffer I (SignalChem) and ATP[γ32P], and incubated for 90 minutes at 30 °C. Each well contains a mixture of peptides with a centralized phospho-acceptor (serine and threonine at a 1:1 ratio) and one fixed amino acid, in a randomized background. All 20 amino acids are sampled across the range of −5 to +4, relative to the centralized serine/threonine, to determine the individual contributions of amino acids along the substrate peptide, as shown in the heatmap where the x-axis represents fixed amino acid and the y-axis represents relative position. The resulting densitometry values are used to generate substrate logos for the kinase. (**d**) Sequence logo of the substrate consensus motif of DYRK1A as determined in (**c**). The central residue “0” on the X axis denotes the phosphoacceptor serine or threonine. “−1” and “1”, for example, denote the immediate N-terminal and C-terminal residues respectively relative to the serine or threonine phosphorylation site. Taking into account how kinases are selective of the surrounding amino acid sequence, the letter height in the logos is proportional to the favorability of the corresponding amino acid at each position.

Folate-mediated one-carbon metabolism is crucial for many biochemical processes, including biosynthesis of purine, dTMP, methionine and SAM^1–3^. The one-carbon unit is carried by the cofactor THF, and can exist in three different oxidation states, including the most oxidized formyl state, the intermediate methylene state, and the most reduced methyl state (Fig. 1a). In the cytosol, 10-formyl-THF and 5,10-methylene-THF can be readily interconverted by methylenetetrahydrofolate dehydrogenase 1 (MTHFD1). By contrast, the NADPH-dependent reduction of 5,10-methylene-THF into 5-methyl-THF, catalyzed by MTHFR, is irreversible in cells, due to the large standard free energy change and high cytosolic NADPH: NADP ratios^4^. Thus, MTHFR commits one-carbon units to methionine and SAM synthesis, and must be tightly regulated to prevent depletion of 10-formyl-THF and 5,10-methylene-THF, which are crucial for purine and dTMP synthesis respectively.

Two types of regulation of MTHFR have been documented. First, MTHFR is allosterically inhibited by the pathway end product SAM (Fig. 1a)^5,6^. This inhibition can be relieved by S-adenosylhomocystein (SAH) in a competitive manner. Second, when expressed in insect cells or human cells, human MTHFR is found to be phosphorylated at up to 11 serine or threonine sites in a serine-rich region^7,8^. This multisite phosphorylation increases the sensitivity of MTHFR to SAM inhibition *in vitro*^7,8^.

Human MTHFR consists of an N-terminal, catalytic domain and a C-terminal, regulatory domain. The N-terminal domain contains the serine-rich phosphorylation region and the catalytic active site. The C-terminal domain binds SAM to exert allosteric inhibition of MTHFR. The recently solved crystal structure of human MTHFR does not contain the N-terminal serine-rich phosphorylation region (aa 1–37). Nevertheless, the crystal structure suggests that the N-terminal phosphorylation region is positioned near the C-terminal SAM-binding site, raising the possibility that the negative regulations of MTHFR by N-terminal phosphorylation and by SAM could be concerted^8^. Specifically, when both the N-terminal phosphorylation and SAM binding are present, MTHFR is maximally shifted to an inactive conformation.

Many questions related to MTHFR phosphorylation remain open. Which kinases phosphorylate these sites in the N-terminal serine-rich region? How is the phosphorylation regulated? What’s the physiological function of the phosphorylation? This study was initiated to address these questions. In particular, regarding the first question, we formulated hypotheses based on our knowledge of protein kinase substrate specificity, and then used a combination of pharmacology and genetics to test these hypotheses.

## Results

### Proposed scheme for the N-terminal multisite phosphorylation of MTHFR

Figure 1b shows our proposed scheme for the multisite phosphorylation of the N-terminal serine-rich region of MTHFR, involving four classes of kinases, DYRKs, GSK3, CK1, and CK2. This scheme was based on substrate motifs of these kinases, which we determined using a positional scanning peptide library approach^9^ (Fig. S1). First, T34 is phosphorylated by DYRKs, which have a strong preference for arginine at −3 position and proline at +1 position relative to the phosphorylation site at T34 (Figs 1c,d and S1)^10^. Next, T34 phosphorylation in turn primes phosphorylation at S30 and S26 by GSK3, which has a strong preference for phosphoserine or phosphothreonine at +4 position relative to its phosphorylation site. S30 phosphorylation then primes phosphorylation at S33 by CK1, which in turn triggers another series of GSK3 phosphorylation at S29, S25, and S21. Finally, S26 and S21 phosphorylation primes S23, S20, and S18 phosphorylation by CK2.

### Mutational analysis of the N-terminal serine-rich region of MTHFR

**Figure 2:**
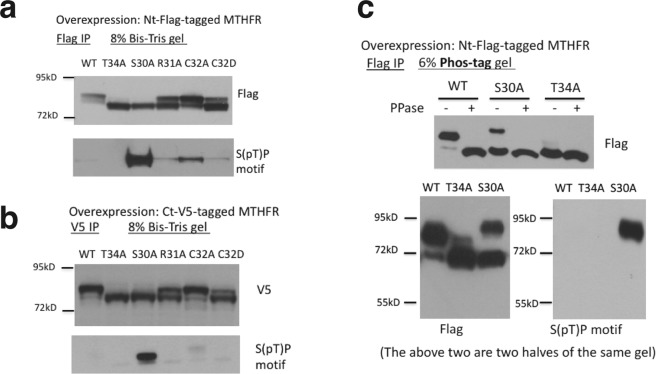
Mutational analysis of the N-terminal serine-rich region of MTHFR in MDA-MB-468 cells. (**a**) Western blot analysis of immunoprecipitated, N-terminal-Flag-tagged MTHFR carrying various mutations, run on 8% Bis-Tris gel. (**b**) The same as (A), but the ectopic V5-tag was located at the C-terminus instead. (**c**) Top, the wild-type (WT), S30A, and T34A MTHFR immunoprecipitates in (**a**) were further analyzed on Phos-tag gels, with or without Calf Intestinal phosphatase (CIP) treatment. Bottom, S(pT)P motif antibody showed reactivity towards the upper, but not the lower, band of S30A MTHFR on phos-tag gel.

Our proposed scheme has three predictions. First, T34 phosphorylation by DYRKs primes all the subsequent phosphorylation events. Indeed, the T34A mutant migrated faster than the wild-type on both regular Bis-Tris and phos-tag gels, and was resistant to calf intestine alkaline phosphatase treatment (Fig. 2a–c), consistent with previous studies^7,11^. Second, R31 is a strong determinant for the priming phosphorylation at T34 by DYRKs. As expected, the R31A mutant was phosphorylated to a much lesser extent than wild-type MTHFR (Fig. 2a,b). Third, the S30A mutation abolishes GSK3 phosphorylation, resulting in a singly phosphorylated species that can be recognized by the S(pT)P motif antibody (Fig. 1b), which importantly does not react with (pS)(pT)P. This was observed (Fig. 2a–c), although it was apparent on phos-tag gel that only ~1/3 of the S30A MTHFR protein was phosphorylated at T34, with the remainder being unphosphorylated (Fig. 2c).

### Evidence for DYRK1A and DYRK2 as priming kinases for MTHFR T34 phosphorylation

**Figure 3:**
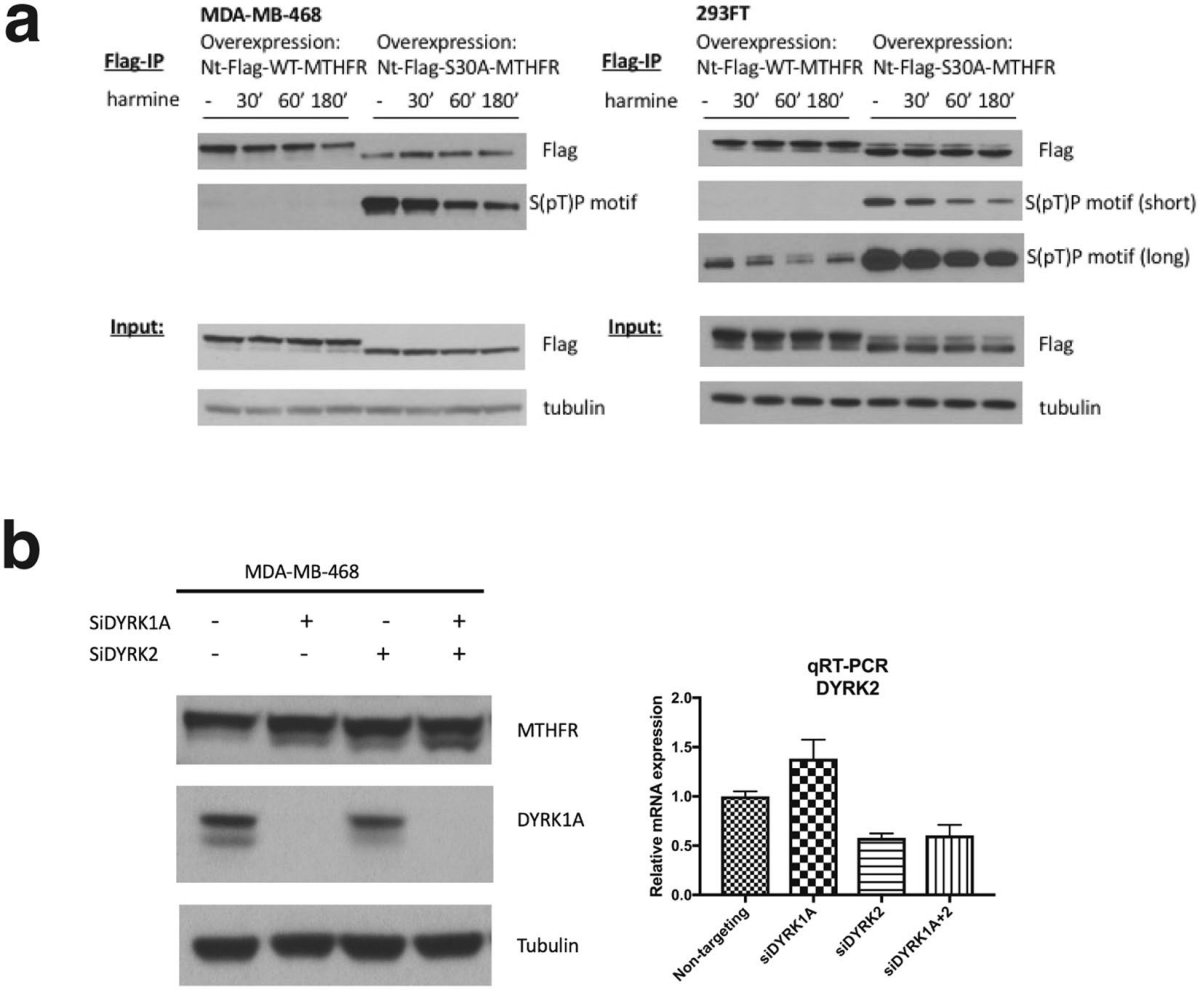
Evidence for DYRK1A and DYRK2 as priming kinases for MTHFR T34 phosphorylation. (**a**) Western blot analysis of immunoprecipitated, N-terminal-Flag-tagged wild-type or S30A-MTHFR from MDA-MB-468 and 293FT cells, treated with 1 μM harmine for the indicated times. (**b**) Western blot analysis of MTHFR from MDA-MB-468 cells treated with various combinations of non-targeting control, siDYRK1A, siDYRK2 for 48 h. The endogenous MTHFR was probed using MTHFR monoclonal antibody 5D3. DYRK2 knockdown was assessed by qRT-PCR analysis. n = 4. Data are represented as mean ± SD.

To test if DYRKs are the priming kinases, we treated both wild-type and S30A-MTHFR overexpressing MDA-MB-468 and 293FT cells with a relatively specific DYRK inhibitor, harmine^12^. Although wild-type MTHFR was largely unaffected, S30A-MTHFR was rapidly dephosphorylated, as indicated by the decrease in intensity of the band recognized by the S(pT)P motif antibody (Fig. 3a). The reason why wild-type MTHFR did not respond to harmine will be discussed in the “Discussion”.

Furthermore, we treated MDA-MB-468 cells with siRNAs against DYRK1A and/or DYRK2. The treatment with siRNAs against both DYRK1A and DYRK2 led to a visible increase in the lower, presumably unphosphorylated band in both cell lines (Fig. 3b). Together, the results suggest that DYRK1A and DYRK2 are responsible for MTHFR T34 phosphorylation. Nevertheless, since the reduction in phosphorylation was incomplete, it is likely that additional kinases also work on this site.

### Evidence for subsequent involvement of GSK3 in MTHFR phosphorylation

**Figure 4:**
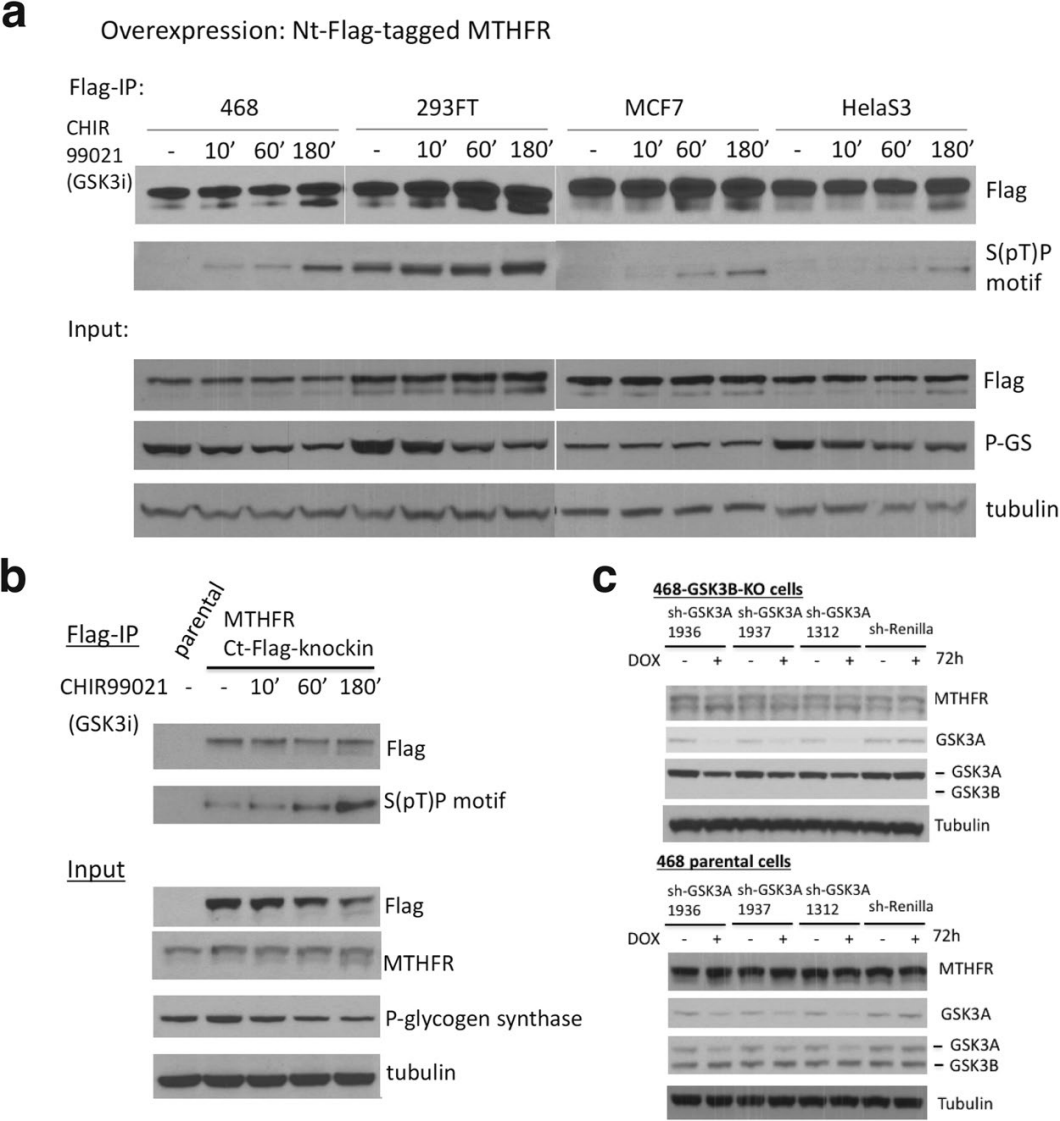
Evidence for subsequent involvement of GSK3 in MTHFR phosphorylation. (**a**) Western blot analysis of immunoprecipitated, N-terminal-Flag-tagged wild-type MTHFR overexpressed from MDA-MB-468, 293FT, MCF7, and HelaS3, treated with 1 μM the GSK3 inhibitor, CHIR99021 for 0, 10 min, 60 min, and 3 h. P-GS denotes phospho-glycogen synthase Ser641, a known site for GSK3^25^. (**b**) The same as (**a**), except that a C-terminal Flag tag was knocked into both alleles of the endogenous *MTHFR* locus using CRISPR. (**c**) Western blot analysis of MDA-MB-468 parental or *GSK3B*-knockout cells, with or without doxycycline-inducible knockdown of GSK3A. DOX concentration was 1 μg/ml.

To test if GSK3 is responsible for subsequent phosphorylations, we treated Flag-tagged MTHFR overexpressing MDA-MB-468, 293FT, MCF7, and HelaS3 cells with the GSK3 inhibitor CHIR99021. Although dephosphorylation was slow, the appearance of a lower Flag-tagged MTHFR band was apparent by 3 h and was accompanied by an increase in intensity of the band recognized by the S(pT)P motif antibody (Fig. 4a). Thus, GSK3 inhibition mimics the effect of S30A mutation (Fig. 2a,b), indicating that S30 is one of the GSK3 phosphorylation sites (Fig. 1b). To rule out overexpression artifacts, using CRISPR we introduced a C-terminal Flag tag into the endogenous MTHFR locus in MDA-MB-468 cells, and the same result was obtained with GSK3 inhibition (Fig. 4b).

To complement pharmacology with genetics, we also tested the effect of inducible knockdown of GSK3A in MDA-MB-468 parental and *GSK3B* CRISPR knockout cells. Whereas knocking down GSK3A in the parental background had no effect, in the *GSK3B*-knockout cells this led to marked reduction in MTHFR phosphorylation, as indicated by the prominence of the lower MTHFR band (Fig. 4c). Together, these results point to the role of both GSK3A and GSK3B in MTHFR phosphorylation.

### MTHFR N-terminal phosphorylation is maintained by SAM binding to the C-terminal regulatory domain

**Figure 5:**
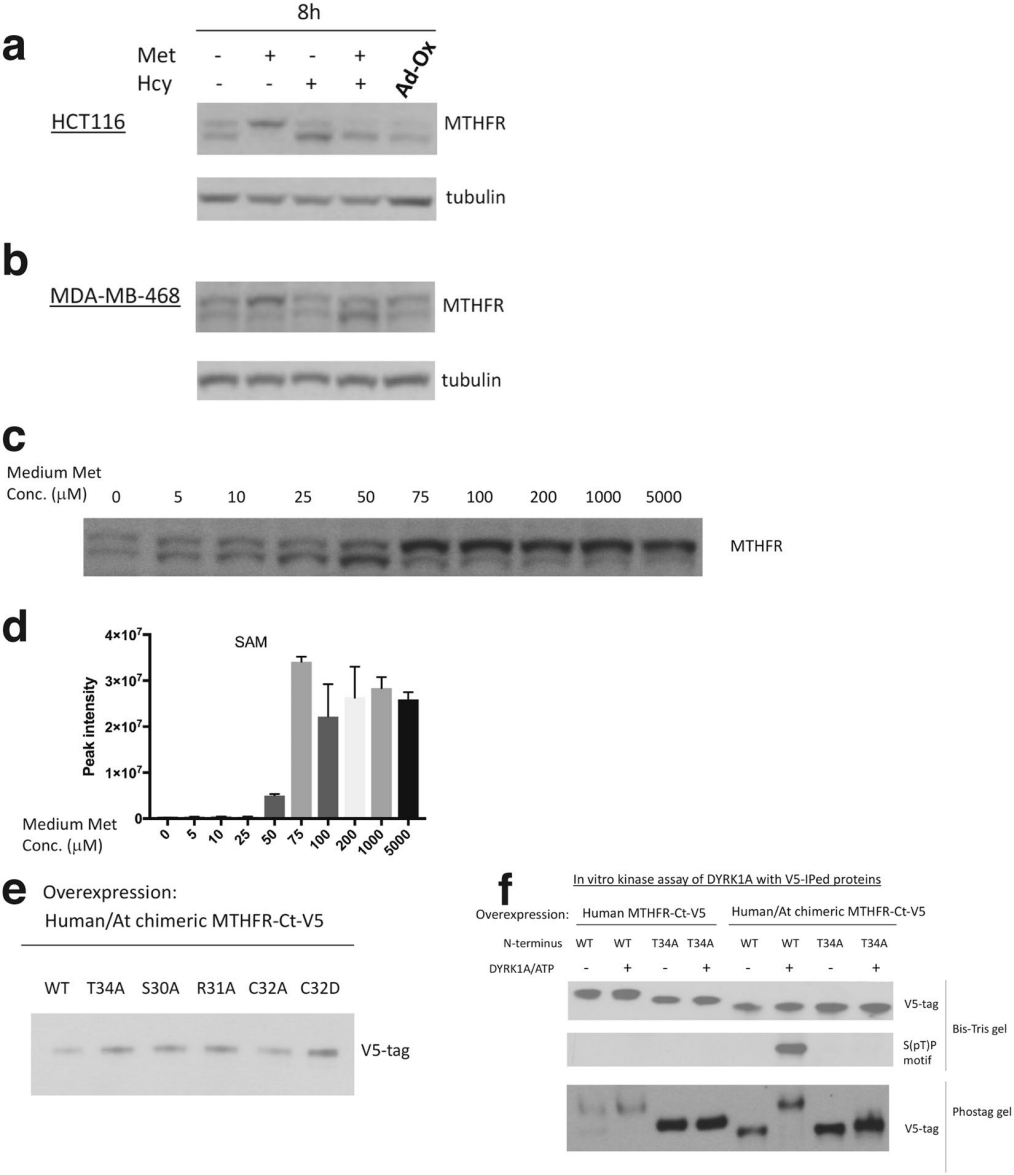
MTHFR N-terminal phosphorylation is maintained by SAM binding to the C-terminal regulatory domain. (**a**,**b**) Western blot analysis of HCT116 (**a**) and MDA-MB-468 (**b**) cells subjected to methionine deprivation, 0.5 mM homocysteine treatment, or both, for 8 h. The cells were also treated with 20 μM adenosine 2′3′-dialdehyde, known to decrease SAM/SAH ratio by inhibiting SAH hydrolase^26^, for 8 h. (**c**) Western blot analysis of MDA-MB-468 cells subjected to decreasing medium methionine concentrations for 24 h. (**d**) LC-MS measurement of SAM in MDA-MB-468 cells as treated in (**c**). (**e**) Western blot analysis of immunoprecipitated, C-terminal-V5-tagged, human/Arabidopsis chimeric MTHFR carrying various mutations in the N-terminal serine-rich region, run on 8% Bis-Tris gel. These chimeric MTHFR were overexpressed in MDA-MB-468 cells. (**f**) *In vitro* kinase assay of DYRK1A performed on various substrates, including wild-type and T34A human MTHFR, and wild-type and T34A human/Arabidopsis chimeric MTHFR. These substrates carry a C-terminal V5 tag, and were immunoprecipitated and eluted with V5 peptide from MDA-MB-468 cells prior to the *in vitro* kinase assay.

Previous studies have suggested that MTHFR phosphorylation can be reduced upon methionine deprivation or homocysteine addition, conditions associated with a decreased cellular SAM/SAH ratio^7,13^. We have confirmed these findings in both HCT116 and MDA-MB-468 cells (Fig. 5a,b). Moreover, we found that a gradual decrease in medium methionine concentration from supraphysiological to subphysiological levels also reduced MTHFR phosphorylation (Fig. 5c). The point at which significant reduction in MTHFR phosphorylation started to occur (at 50 μM medium methionine) coincided with a sudden drop in intracellular SAM levels (Fig. 5d), suggesting that adequate SAM levels are crucial for maintaining MHTFR phosphorylation.

Because human MTHFR has a C-terminal regulatory domain that binds SAM, we questioned whether this domain is essential for sensing SAM levels and sustaining MTHFR phosphorylation. To this end, we engineered a chimeric MTHFR where the human N-terminal domain is fused to the Arabidopsis C-terminal domain that is known to be insensitive to SAM regulation^14^. Unlike human MTHFR, this chimeric MTHFR and its various point mutants did not display differences in mobility on SDS-PAGE (cf. Figs 5e with 2a,b), suggesting lack of phosphorylation.

To further confirm the lack of phosphorylation for the chimeric protein, we performed an *in vitro* kinase assay where purified DYRK1A was allowed to react with immunoprecipitated human or chimeric MTHFR in the presence of ATP. For human MTHFR, there was little reaction because the majority of the protein was already phosphorylated. By contrast, the chimeric MTHFR with the wild-type, but not T34A, serine-rich region was efficiently phosphorylated by DYRK1A, as indicated by the s(pT)P motif band and by the band shift on the phos-tag gel (Fig. 5f). Together, these results suggest that maintenance of mammalian MTHFR N-terminal phosphorylation is critically dependent on SAM binding to the C-terminal regulatory domain, presumably due to the protection of these sites from dephosphorylation when bound to the C-terminal regulatory domain that is occupied by SAM.

### Wild-type and non-phosphorylatable mutant MTHFR respond to homocysteine differently

**Figure 6:**
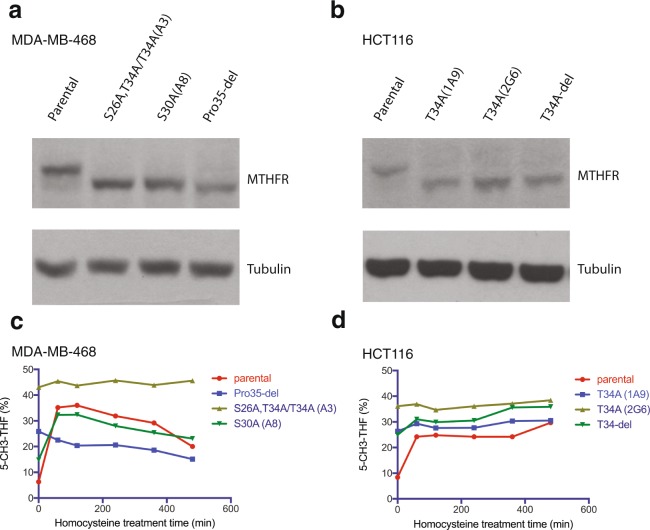
Wild-type and non-phosphorylatable mutant MTHFR respond to homocysteine differently. (**a**) Western blot analysis of MDA-MB-468 parental and MTHFR CRISPR knockin mutant cells. For the A3 clone, one MTHFR allele is S26A and T34A, and the other MTHFR allele is T34A. All the other clones carry homozygous MTHFR mutations. (**b**) Western blot analysis of HCT116 parental and MTHFR CRISPR knockin mutant cells. All the clones carry homozygous MTHFR mutations. (**c**,**d**) Changes in 5-CH_3_-THF levels in response to 0.5 mM homocysteine treatment for the isogenic lines of MDA-MB-468 (**c**) and HCT116 (**d**). 5-CH_3_-THF was measured by HPLC and quantified as percentage of total folates.

Phosphorylated MTHFR is less active than unphosphorylated MTHFR at physiologically relevant SAM concentrations (1–3 μM) *in vitro*^8^. To confirm this in cells, using CRISPR we knocked in non-phosphorylatable mutations at the endogenous MTHFR locus in MDA-MB-468 and HCT116 cell lines (Fig. 6a,b), and compared their folate response to homocysteine treatment. Most of these knockin cells proliferate at rates comparable to the parental lines (Fig. S2). For both parental lines, homocysteine addition caused a dramatic increase in 5-CH_3_-THF production (Fig. 6c,d). By contrast, the knockin cell lines with non-phosphorylatable MTHFR had high basal levels of 5-CH_3_-THF and did not respond to homocysteine stimulation (Fig. 6c,d). For MDA-MB-468 S30A knockin (which partially retains T34 phosphorylation, Fig. 2), homocysteine addition caused an increase in 5-CH_3_-THF, but the magnitude of increase was less than that of MDA-MB-468 parental cells (Fig. 6c). Thus, wild-type MTHFR became more active as the SAM/SAH ratio decreased and as its phosphorylation was reduced, both as a result of homocysteine treatment. These results suggest that MTHFR can be switched on through dephosphorylation, to control the level of homocysteine by providing the methyl group for homocysteine remethylation.

## Discussion

**Figure 7:**
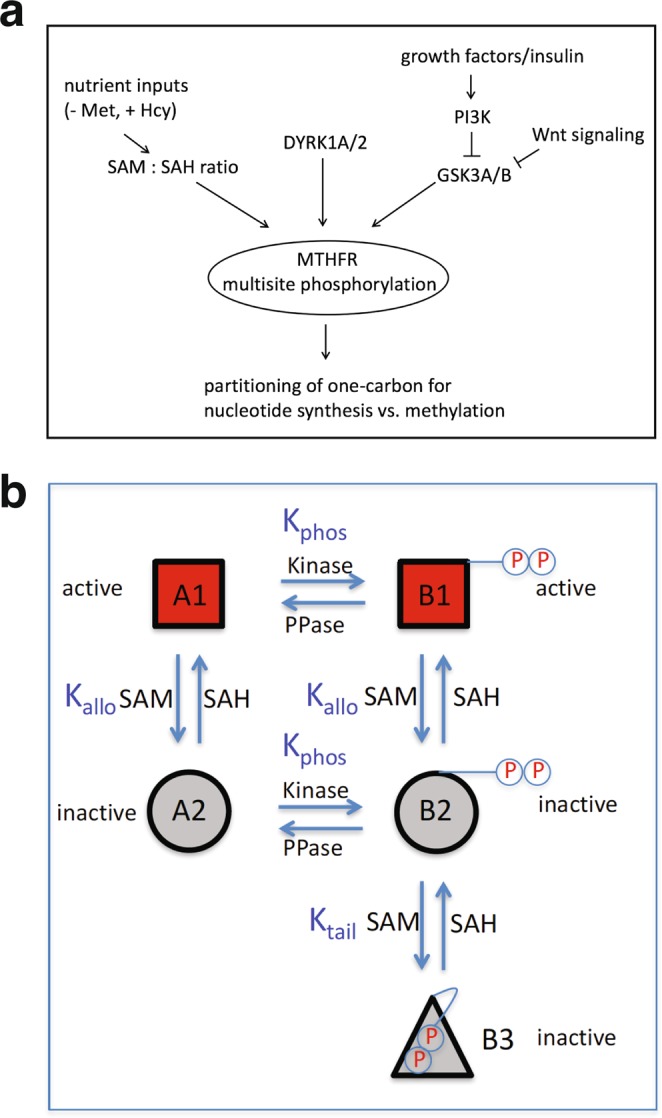
Models. (**a**) MTHFR multisite phosphorylation integrates various signals, such as nutrient inputs that influence the cellular SAM:SAH ratio, and signaling pathways that modulates DYRK1A and GSK3 kinase activities. (**b**) A molecular model explaining why MTHFR phosphorylation responds to changes in the SAM/SAH ratio, and why multiply phosphorylated MTHFR is more sensitive to SAM inhibition. In this model, multisite phosphorylation of the N-terminal tail of MTHFR allows a new inactive conformation (B3), where the N-terminal tail is not accessible for phosphatases. When SAM level is high, SAM can preferentially bind to and stabilize the inactive states A2, B2, and B3, thereby shifting the equilibrium to these inactive states including B3 that cannot be dephosphorylated. Conversely, when SAH level is high, the action of SAM is reversed, thereby favoring conformations (B1 and B2) that can be readily dephosphorylated. Multiply phosphorylated MTHFR is more sensitive to SAM inhibition because, unlike unphosphorylated MTHFR, it can be further trapped in the inactive, B3 state.

In this study, we identified DYRK1A/2 and GSK3A/B among the kinases that phosphorylate the N-terminal serine-rich region of MTHFR (Fig. 7a). Both of these kinases are known to be constitutively active in resting cells^15,16^, thus explaining why the majority of MTHFR is phosphorylated under basal conditions. However, these kinases could be inhibited under certain conditions, thereby altering the degree of MTHFR phosphorylation. For example, GSK3 is known to be inhibited by PI3K-Akt signaling^17^, so the level of MTHFR phosphorylation might be reduced under conditions of PI3K hyperactivation, which occurs often in cancers. By contrast, the regulation of DYRK1A/2 is poorly understood^15^. DYRK1A is overexpressed in Down Syndrome, and DYRK1A overexpression in mice led to perturbations of the methionine cycle, specifically a decrease in plasma homocysteine level^18^. Such an effect of DYRK1A overexpression is not predicted by our model, where DYRK1A-primed MTHFR phosphorylation would lead to a decrease in MTHFR activity, hence an increase in homocysteine.

We confirmed previous findings that MTHFR N-terminal phosphorylation is also regulated by the cellular SAM/SAH ratio (Fig. 7a)^7,13^. Furthermore, we provided evidence that this regulation requires the C-terminal SAM binding domain. Such interaction between MTHFR N-terminal phosphorylation region and the C-terminal SAM-binding domain is well-supported by the recently solved crystal structure of human MTHFR, which suggests proximity of these two regions^8^. Presumably, when SAM is bound, the N-terminal phosphorylation region interacts with the C-terminal domain, such that the multiple phosphorylation sites are buried and therefore shielded from phosphatases (Fig. 7b). The strength of this interaction might depend on the number of phosphates. If so, this could explain why the DYRK1A inhibitor harmine failed to dephosphorylate wild-type MTHFR but worked efficiently on the singly phosphorylated S30A species (Fig. 3a). On the other hand, in the absence of SAM or when SAH instead is bound, this interaction is weak or absent, so the phosphorylation sites are exposed to phosphatases, leading to dephosphorylation.

The model presented in Fig. 7b can also explain why the multisite phosphorylation increases the sensitivity to SAM inhibition. There are five distinct conformation states: the top two are active, the middle two are inactive, and the bottom one is also inactive. SAM preferentially binds to and stabilizes the middle two inactive conformations. When MTHFR is multiply phosphorylated, however, SAM binding further shifts the middle inactive state towards the bottom inactive state. Thus, in the presence of SAM, the proportion of inactive states is higher than without the multisite phosphorylation.

What’s the physiological function of MTHFR multisite phosphorylation? We surmise that under high methionine conditions, the constitutive, multisite phosphorylation ensures that MTHFR is maximally inhibited by SAM, so that one-carbon units can be spared for crucial processes such as purine and dTMP synthesis. Under low methionine conditions, MTHFR is dephosphorylated and becomes more active (Fig. 5c), so that more one-carbon units are diverted to produce methionine and SAM. In addition, our results with homocysteine-treated cells (Fig. 6c,d) suggest that MTHFR can be dephosphorylated and disinhibited to provide 5-CH_3_-THF for homocysteine remethylation. High concentrations of homocysteine are toxic, posing risk for cardiovascular diseases^19^. Thus, MTHFR dephosphorylation and disinhibition may also represent a cellular response to ameliorate hyperhomocysteinemia.

## Methods

### Cell lines

MDA-MB-468, MCF7, HeLaS3 were purchased from ATCC. 293FT and HCT116 were lab stocks. All these cell lines were cultured in DMEM with 10% FBS.

### Antibodies and small molecule inhibitors

MTHFR monoclonal antibody 5D3 was purchased from Sigma-Aldrich (Cat# SAB5300417). SpTP motif, DYRK1A, GSK3A/B, GSK3A, P-glycogen synthase antibodies was purchased from Cell Signaling Technology (Cat# 5243, Cat# 2771, Cat#5676, Cat#4818, Cat#94905 respectively).

DYRK inhibitor harmine was purchased from Sigma-Aldrich (Cat# 286044). GSK3 inhibitor CHIR-99021 was purchased from Selleckchem (Cat# S2924).

### Peptide library approach for determining kinase phosphorylation Motifs

Reagents used for the peptide library experiments include: Kinase substrate library (Anaspec). Streptavidin conjugated membranes (Promega). Recombinant kinases were obtained from the following sources: DYRK2 (SignalChem), GSK3A (SignalChem), GSK3B (SignalChem), CK1G (SignalChem), CK1E (SignalChem), CK1A (ThermoFisher Scientific), CK2A1 (NEB), and DYRK1A (gift from the Matthias Geyer Laboratory).

To determine the phosphorylation motifs for the kinases, *in vitro* kinase assays were performed with recombinant kinases on the kinase substrate library in the presence of ATP[γ-32P]. There reactions were carried out in Kinase Buffer I (SignalChem) at 30 °C for 90 minutes. The peptides, which contain C-terminal biotinyl groups, were blotted onto streptavidin-conjugated membranes and imaged with a Typhoon FLA 7000. Detailed information of the experimental protocol is provided elsewhere^20^.

The spot densitometry values were used to generate sequence logos. The densitometry matrix was normalized by each row, in order to create a probability matrix, was converted to a probability sequence logo. All the sequence logos were generated in R 3.5.1 using the ‘ggseqlogo’ package^21^.

### CRISPR/Cas9-mediated genome-editing in cell lines

Single guide RNA sequence was cloned into PX459_V2.0 (Addgene) according to a published protocol^22^. Cell lines with transfected with guide using Lipofectamine 3000 or Lipofectamine LTX (Invitrogen). 24 hr post transfection, puromycin (typically at 1 μg/ml) was added to enrich positively transfected cells. The puromycin selection typically lasts for 2 days, until mock-transfected, control cells were completely eliminated by puromycin. Single cell cloning was performed by serial dilution in 96-well plates. After 1.5–3 weeks, 15–20 clones were picked for knockout verification.

If antibodies were available, knockout clones were first screened by western blotting. The genotypes of selected clones were determined by PCR amplification of a genomic region encompassing the editing site, TOPO cloning of the individual alleles into PCR4-TOPO vector (Invitrogen), and Sanger sequencing. At least 20 bacterial colonies were picked for sequencing to ensure approximately equal representation of both alleles. We were unable to find a specific DYRK2 antibody, so DYRK2 knockout status was determined by TOPO cloning followed by Sanger sequencing.

MTHFR CRISPR Knockin cells was generated using nucleofection of sgRNAs together with ssODN repair templates. The PAM sequence was mutated and a restriction site was introduced into the ssODN repair templates. Correct clones were initially screened using restriction digest, and further verified using Sanger sequencing of the genomic DNA PCR amplicon.

### siRNA and inducible shRNA knockdown

Pooled siGENOME siRNA (30 nM) against DYRK1A and DYRK2 were purchased from Dharmacon and transfected using Lipofectamine RNAiMAX reagent.

DOX-inducible GSK3A knockdown target sequence was predicted using the Splash algorithm (http://splashrna.mskcc.org/) and cloned into the inducible lentiviral vector according to a previously published protocol^23^. Lentivirus-infected, GFP-positive cells were sorted prior to analysis.

### Cloning of expression constructs

MGC cDNA clones of human MTHFR was purchased from Open Biosystems. Subsequently MTHFR was subcloned into gateway destination vectors, including retroviral MSCV-N-Flag-HA-IRES-PURO (addgene #41033) which contains a N-terminal Flag tag, and lentiviral pLEX_306 (addgene #41391) which contains a C-terminal V5 tag.

The cDNA for Human/Arabidopsis MTHFR chimera was synthesized by IDT. The coding region contains the first 357 amino acids of human MTHFR, followed by KNEE, followed by the last 266 amino acids of Arabidopsis MTHFR. The cDNA was then subcloned into pLEX_306 using gateway technology.

### Generation of lentiviral or retroviral stable cell lines

For lentivirus generation, 293T cells were co-transfected with the viral plasmid of interest together with pVSVG and delta8.9 using Lipofectamine 3000. For retrovirus generation, Phoenix-Ampho cell were transfected with the viral plasmid using Lipofectamine 3000. Lentivirus or retrovirus-containing supernatant was collected at 48 hr after transfection. Target cells were infected with 0.45 μm PVDF membrane-filtered viral supernatant in the presence of 8 μg/ml polybrene for 24 hr and then replaced in fresh media. At about 48–72 hr post-infection, puromycin (typically at 2 μg/ml) was added to select positively transduced cells for at least 3 days.

### Immunoprecipitation

Flag- or V5-tagged proteins were immunoprecipitated using anti-Flag M2 agarose (Sigma-Aldrich, Cat# A2220) or anti-V5 agarose (Sigma-Aldrich, Cat# A 7345), according to manufacturer’s instructions.

### Western blot analysis

Cells were quickly washed twice with ice-cold PBS, and lysed in NP-40 lysis buffer (50 mM Tris-HCl, pH 8.0, 150 mM NaCl, 1.0% NP-40) freshly supplemented with protease inhibitors (Roche or Thermo). Lysates were cleared by centrifugation at 13,000 g at 4 °C for 10 min. Protein concentration was determined by BCA assay. Proteins were denatured in NuPAGE LDS 4X sample buffer (Invitrogen) supplemented with β-mercaptoethanol. Typically, 20–50 μg of total protein was loaded per lane on an 8% Bis-Tris Bolt gel (Invitrogen), and the apparatus was kept on ice, for a 2–3 hour run at 120 volts. 7.5% SuperSep Phos-tag gels were run according manufacturer’s protocols (Wako Cat# 198–17981); in particular, 10 mM MnCl_2_ was included in each sample, and EDTA washes were performed before proceeding to the transfer step. SDS-PAGE was followed by standard immunoblotting steps. Immunoblot signals were detected by enhanced chemiluminescence.

### DYRK1A protein purification and ***In vitro*** kinase assay

For DYRK1A protein expression, One-shot *E.coli* BL21(DE3) (Invitrogen) was transformed with DYRK1A-pNIC28 vector (Addgene, Cat# 38913). The bacteria were grown at 37 °C in 2xYT medium containing 100 μg/ml Carbenicillin until A600nm reached ~0.6, cooled down to room temperature, followed by 0.5 mM IPTG induction of protein expression at room temperature for 16 hours. The DYRK1A protein was purified using the standard protocol for Ni-NTA purification under native conditions as described in “The QIAexpressionist Handbook”.

For DYRK1A *in vitro* kinase assay, immunoprecipitated human or chimeric, wild-type or T34A, MTHFR was incubated with 3 μg of DYRK1A in a kinase assay buffer (50 mM HEPES, pH7.3, 10 mM MgCl_2_, 10 mM MnCl_2_, 1 mM DTT, and 100 μM ATP) at 30 °C for 90 min. The reaction was stopped by adding Invitrogen LDS 4X sample buffer.

### LC-MS analysis of SAM and SAH

Pre-cooled 80% methanol with 0.1% formic acid (1 mL) was added to each 6-cm dish of 70% confluent cells. The lysate was scraped into an Eppendorf tube and then centrifuged at 4 °C for 10 minutes at 14,000 rpm. The supernatant was evaporated using a Speed-Vac. Targeted LC/MS analyses were performed on a Q Exactive Orbitrap mass spectrometer (Thermo Scientific) coupled to a Vanquish UPLC system (Thermo Scientific). The Q Exactive operated in positive mode. An XBridge amide column (2.1 mm i.d. × 150 mm, Waters) was used for separation of metabolites. The flow rate was set at 150 μL/min. Mobile phases consisted of 0.1% formic acid in acetonitrile for line A, and 0.1% formic acid in water for line B. Gradient ran from 70% to 0% A in 13 min followed by a wash with 0% A and re-equilibration at 70% A. Metabolites were identified on the basis of exact mass within 5 ppm and retention time. Relative metabolite quantitation was performed based on peak area for each metabolite. Data analysis was done using in-house written scripts. SAM and SAH metabolites were also manually checked using Thermo X-Caliber. Normalization was based on protein content.

### HPLC analysis for intracellular folates

HPLC analysis of intracellular folates was performed as previously reported^24^. Briefly, cells were cultured in 50 nM ^3^H-5-formyl-tetrahydrofolate for 48 hr. Then cellular folates were liberated from proteins and protected from air oxidation by boiling in 50 mM Tris-HCl pH7.4 buffer with 3% (w/v) ascorbate and 200 μM β-mercaptoethanol. The polyglutamate tails of folates were then cleaved by recombinant γ-glutamyl hydrolase (GGH), and the resulting folate monoglutamates were resolved by reversed phase ion-pair HPLC with radioactivity detection.

## Supplementary information

Supplementary info
