# Supplementary material for: Regulation of folate and methionine metabolism by multisite phosphorylation of human methylenetetrahydrofolate reductase
Source: Sci Rep. 2019 Mar 12;9:4190. doi: 10.1038/s41598-019-40950-7 (PMC6414673; doi:10.1038/s41598-019-40950-7)
Supplement: Supplementary file 1 — Supplementary info [file 41598_2019_40950_MOESM1_ESM.pdf]

Supplementary Information

Regulation of folate and methionine metabolism by multisite phosphorylation of human methylenetetrahydrofolate reductase

Yuxiang Zheng[1], Shivan Ramsamooj[1], Qian Li[1], Jared L. Johnson[1], Tomer M. Yaron[1,2], Klaus Sharra[1], Lewis C. Cantley[1*]
1: Meyer Cancer Center, Weill Cornell Medicine
2: Institute for Computational Biomedicine, Department of Physiology and Biophysics, Weill Cornell Medicine
*Correspondence: LCantley@Med.cornell.edu

Supplemental Figure Legends

Figure S1: Sequence logos of substrate consensus motifs of DYRK1A, DYRK2, GSK3A, GSK3B, CK1A, CK1E, CK1G, and CK2A1, as determined by the peptide library approach. The central residue “0” on the X axis denotes the phosphoacceptor serine or threonine. “-1” and “1”, for example, denote the immediate N-terminal and C-terminal residues respectively relative to the serine or threonine phosphorylation site. Taking into account how kinases are selective of the surrounding amino acid sequence, the letter height in the logos is proportional to the favorability of the corresponding amino acid at each position.

Figure S2: Proliferation curves of MDA-MB-468 (a) and HCT116 (b) parental and CRISPR knockin mutant cells. The cells were seeded at ~5000 cells per well in a 96-well plate, 2 h before imaging by Incucyte. For each cell group, 10 replicate wells were used. The percentage confluence of each well was measured by Incucyte every 6 h.

Figure S1

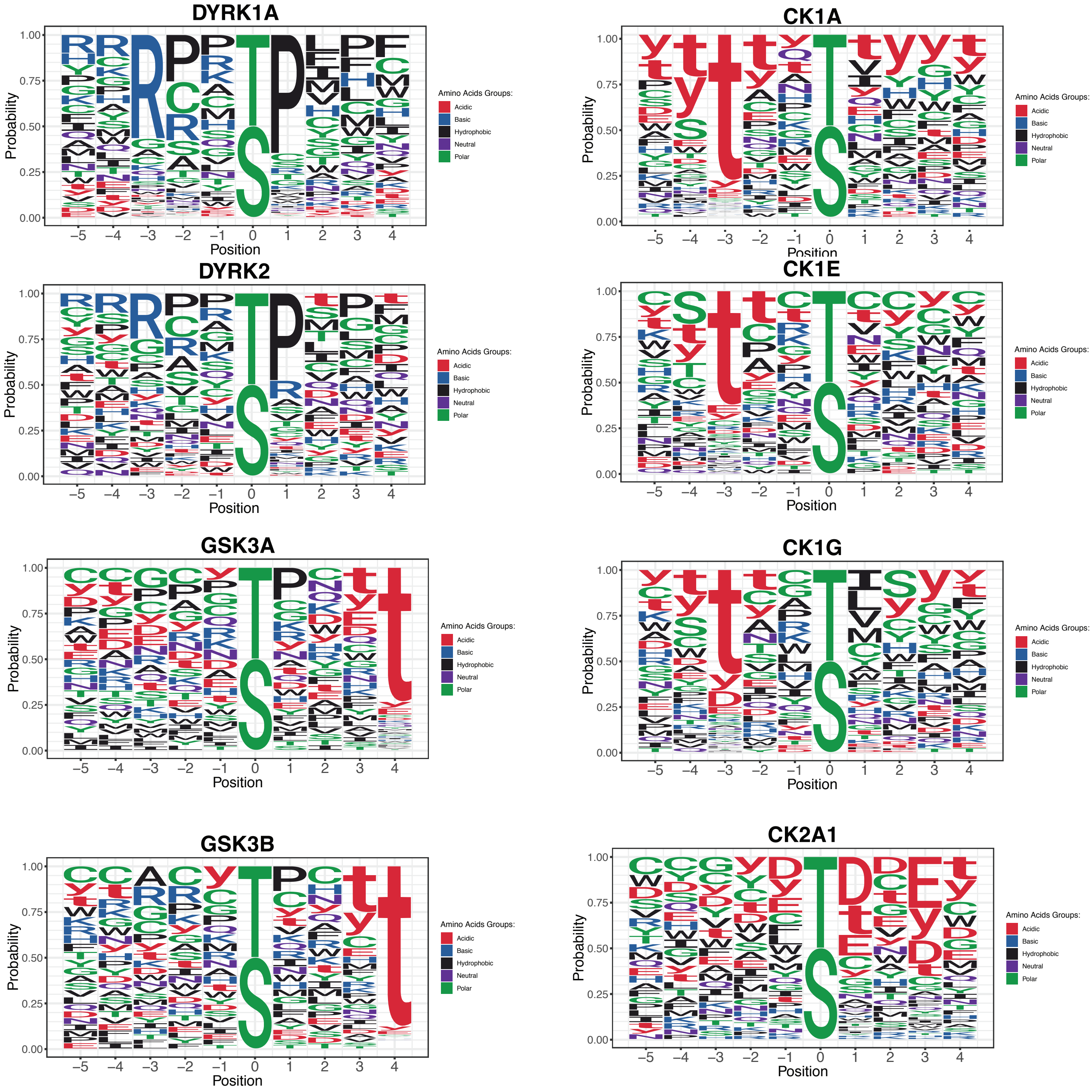

t = phosphothreonine/phosphoserine

y = phosphotyrosine

Figure S2

a

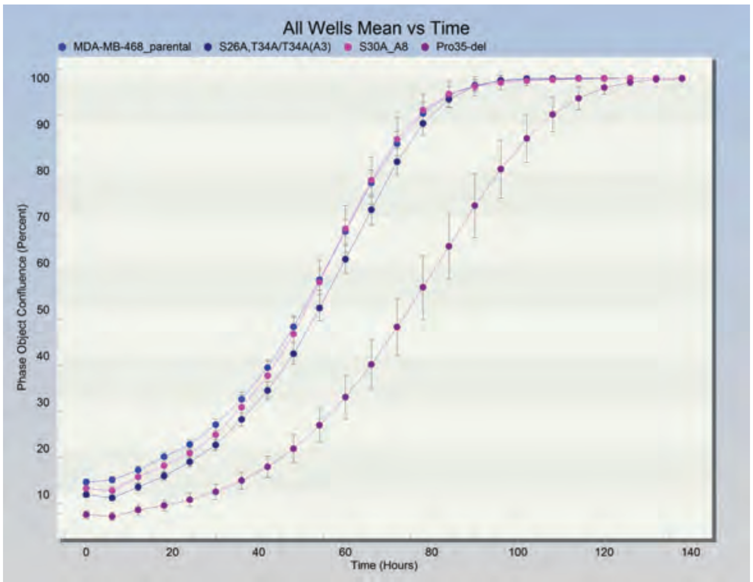
All Wells Mean vs Time
MDA-MB-468_parental
S26A,T34A/T34A(A3)
S30A_A8
Pro35-del
Phase Object Confluence (Percent)
Time (Hours)

b

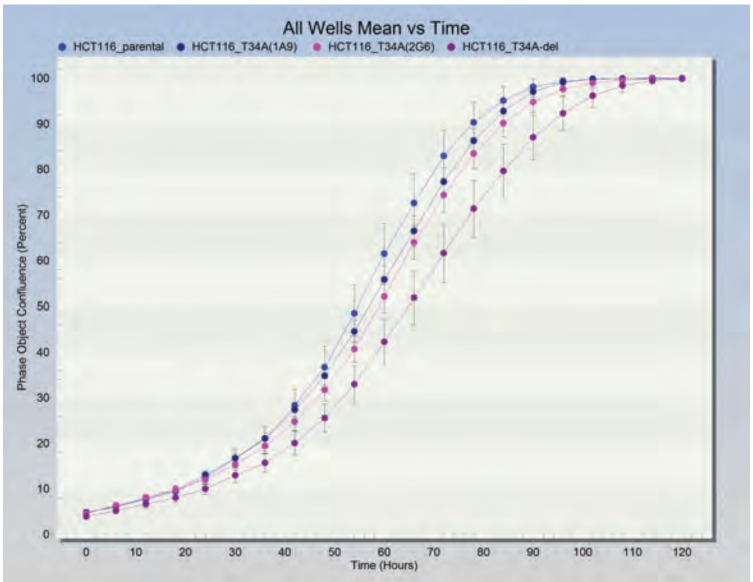
All Wells Mean vs Time
HCT116_parental
HCT116_T34A(1A9)
HCT116_T34A(2G6)
HCT116_T34A-del
Phase Object Confluence (Percent)
Time (Hours)

Figure 2a, top

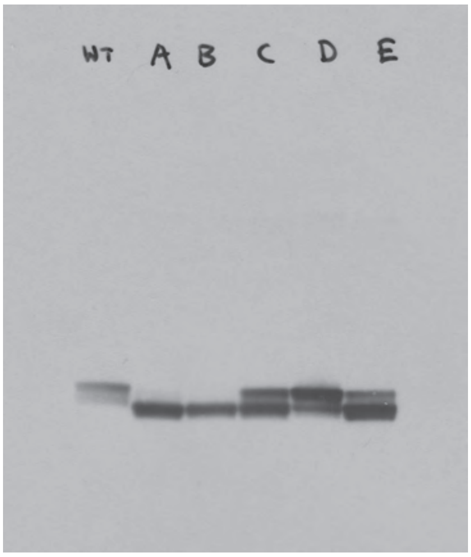

Figure 2a, bottom

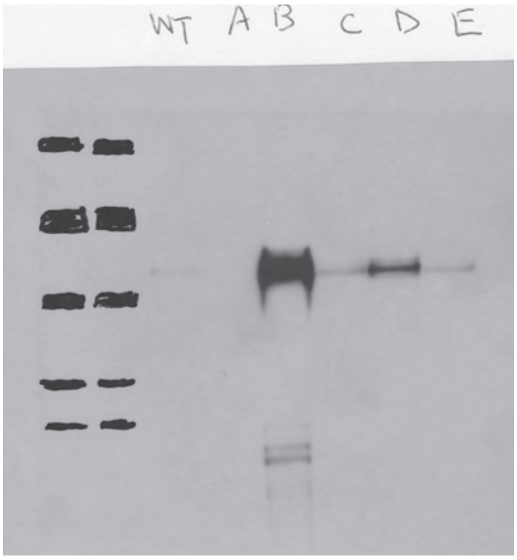

Figure 2b, top

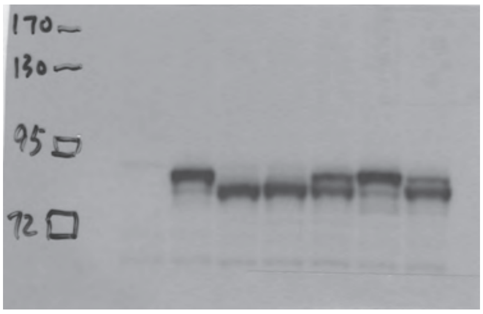

Figure 2b, bottom

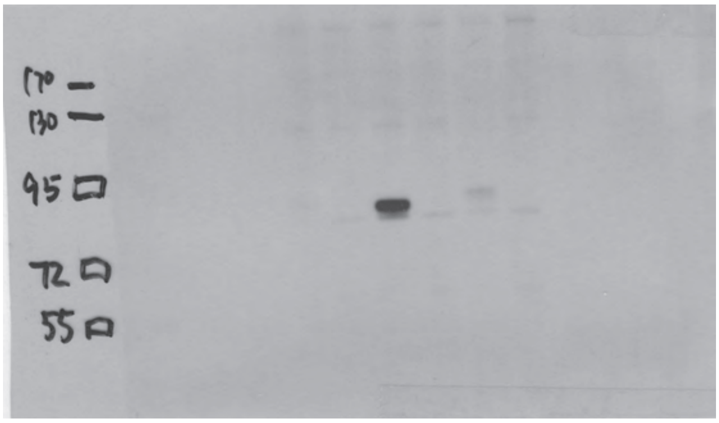

Figure 2c, top

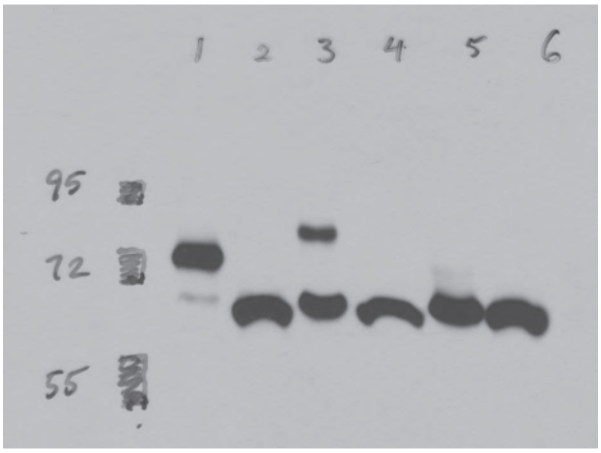

Figure 3, part 1

Figure 3a

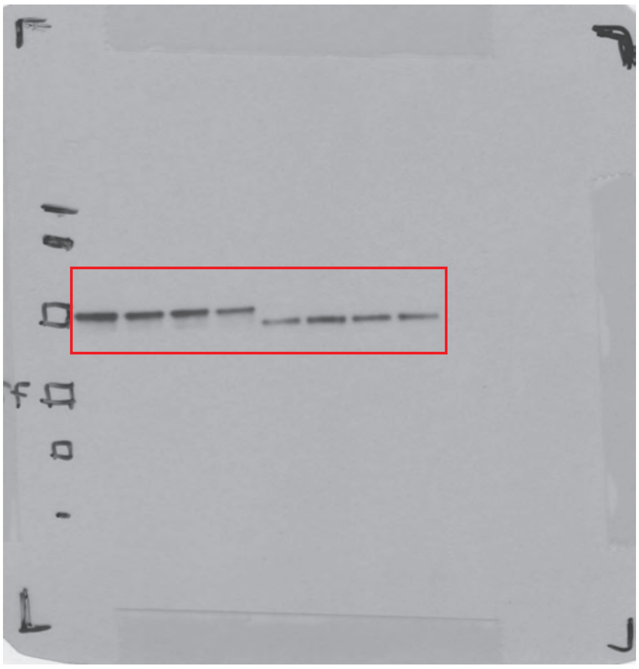

Figure 3a

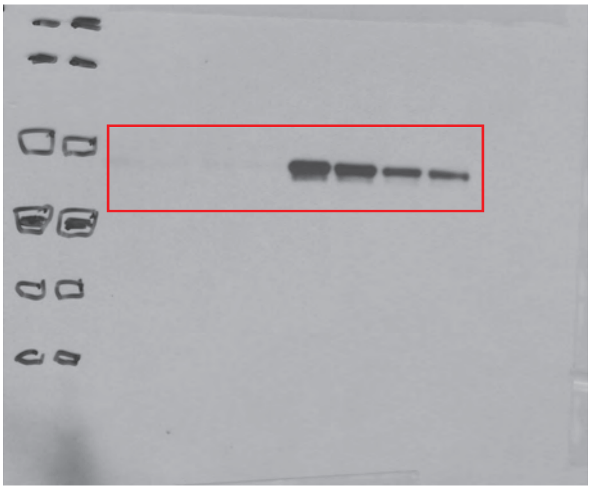

Figure 3a

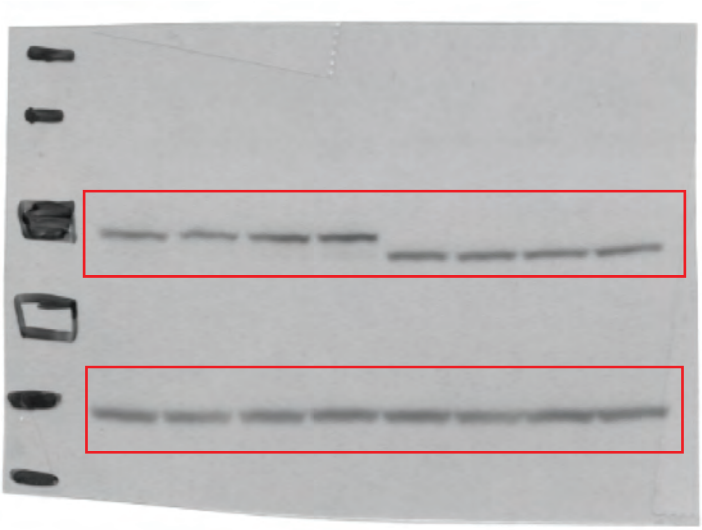

Figure 3, part 2

Figure 3a

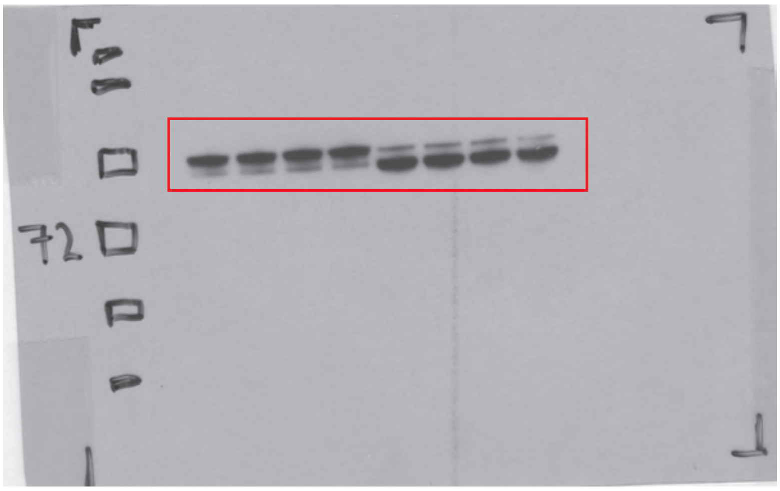

Figure 3a

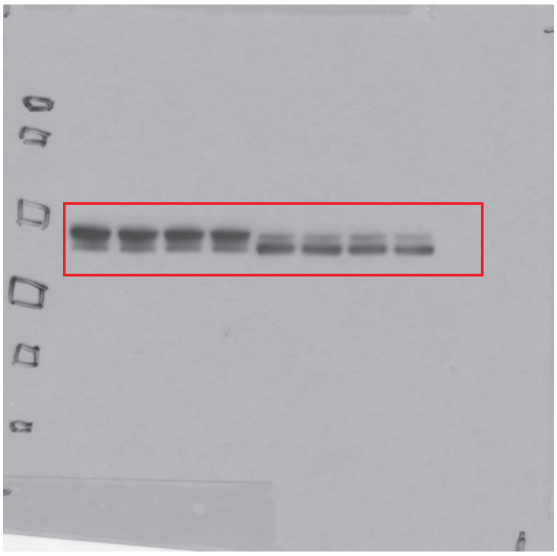

Figure 3a

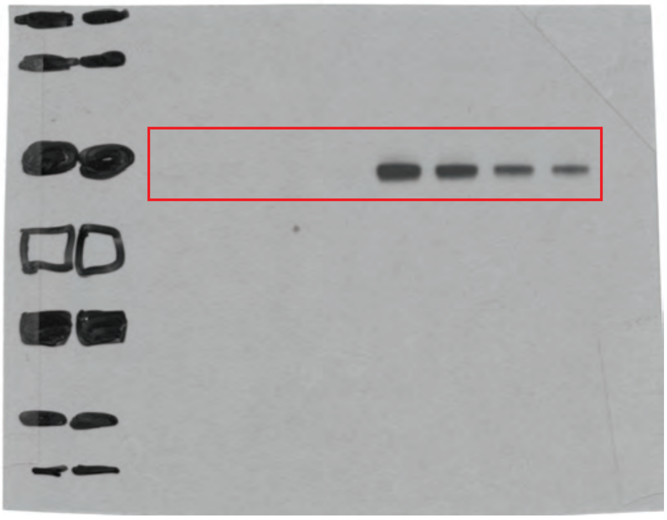

Figure 3a

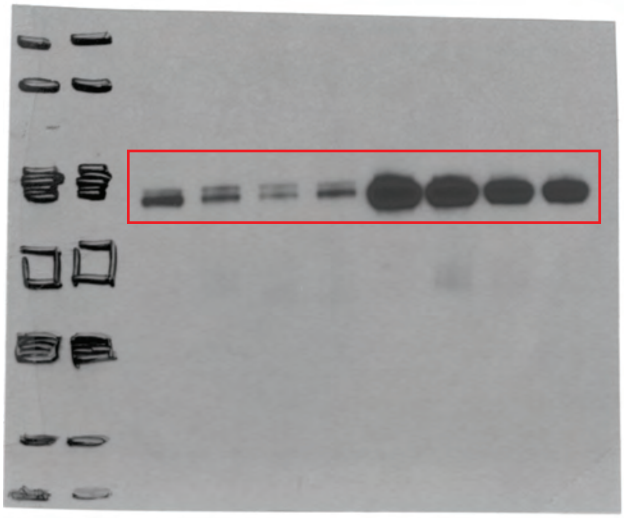

Figure 3a

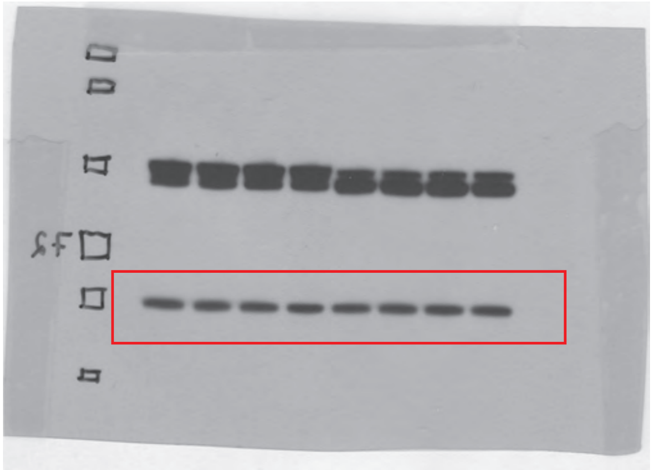

Figure 3, part 3

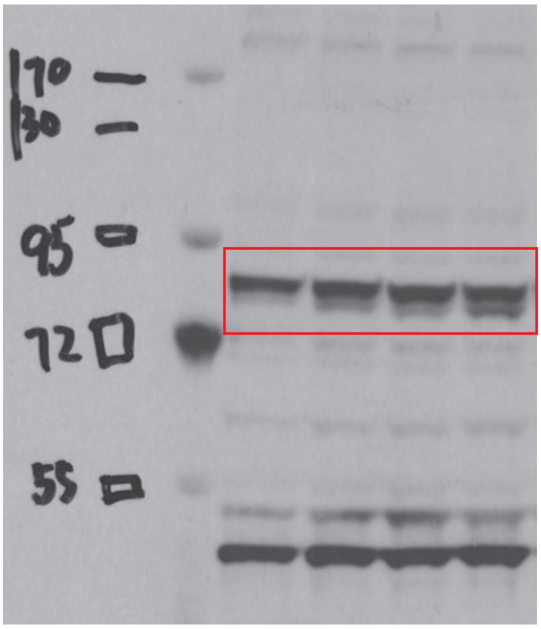

Figure 3b

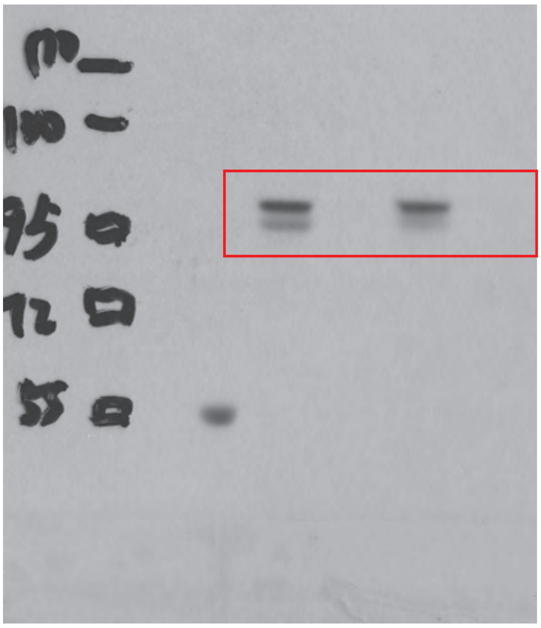

Figure 3b

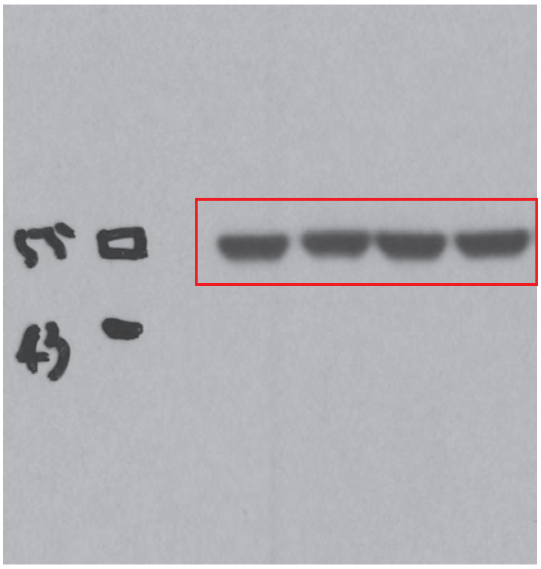

Figure 3b

Figure 4, part 1

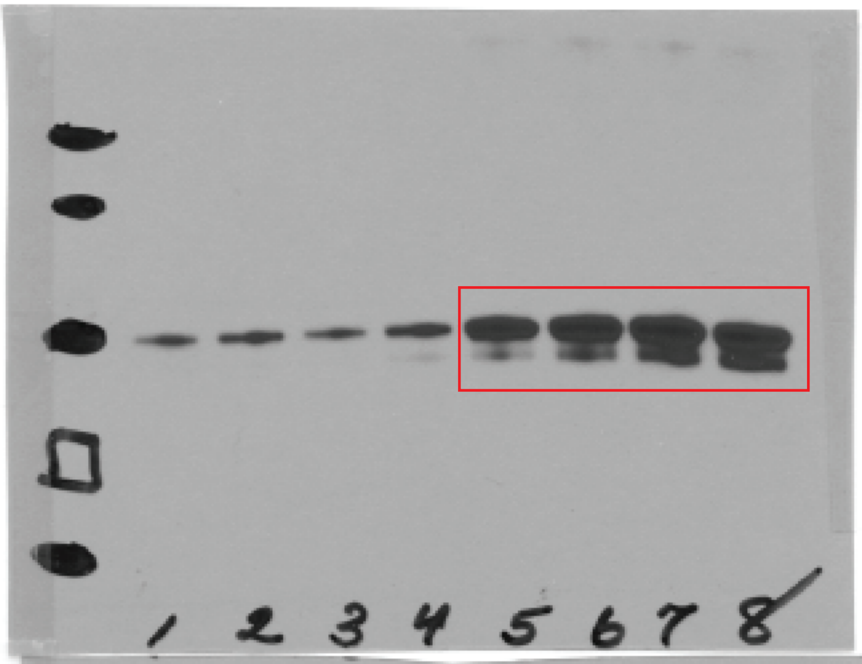

Figure 4a

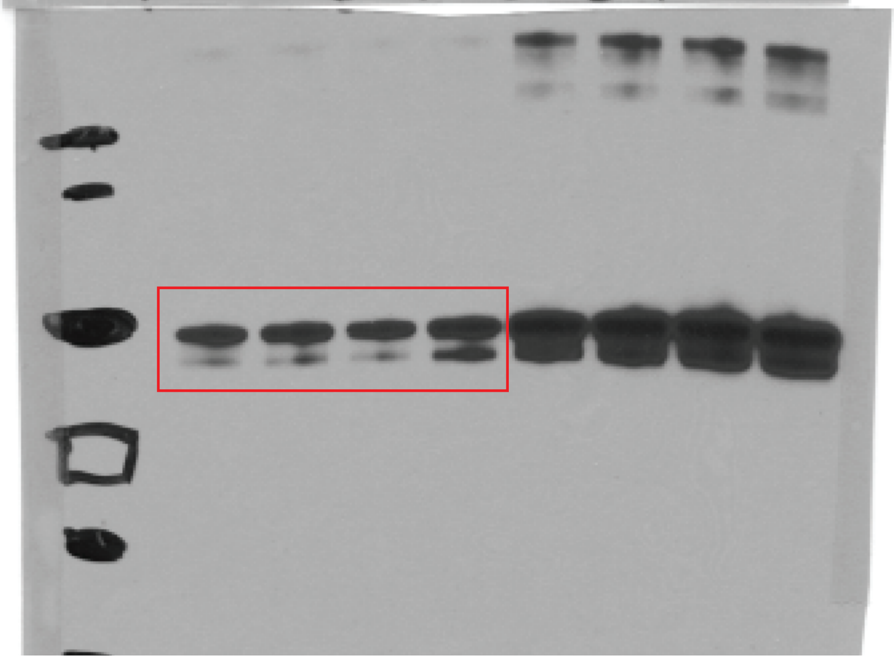

Figure 4a

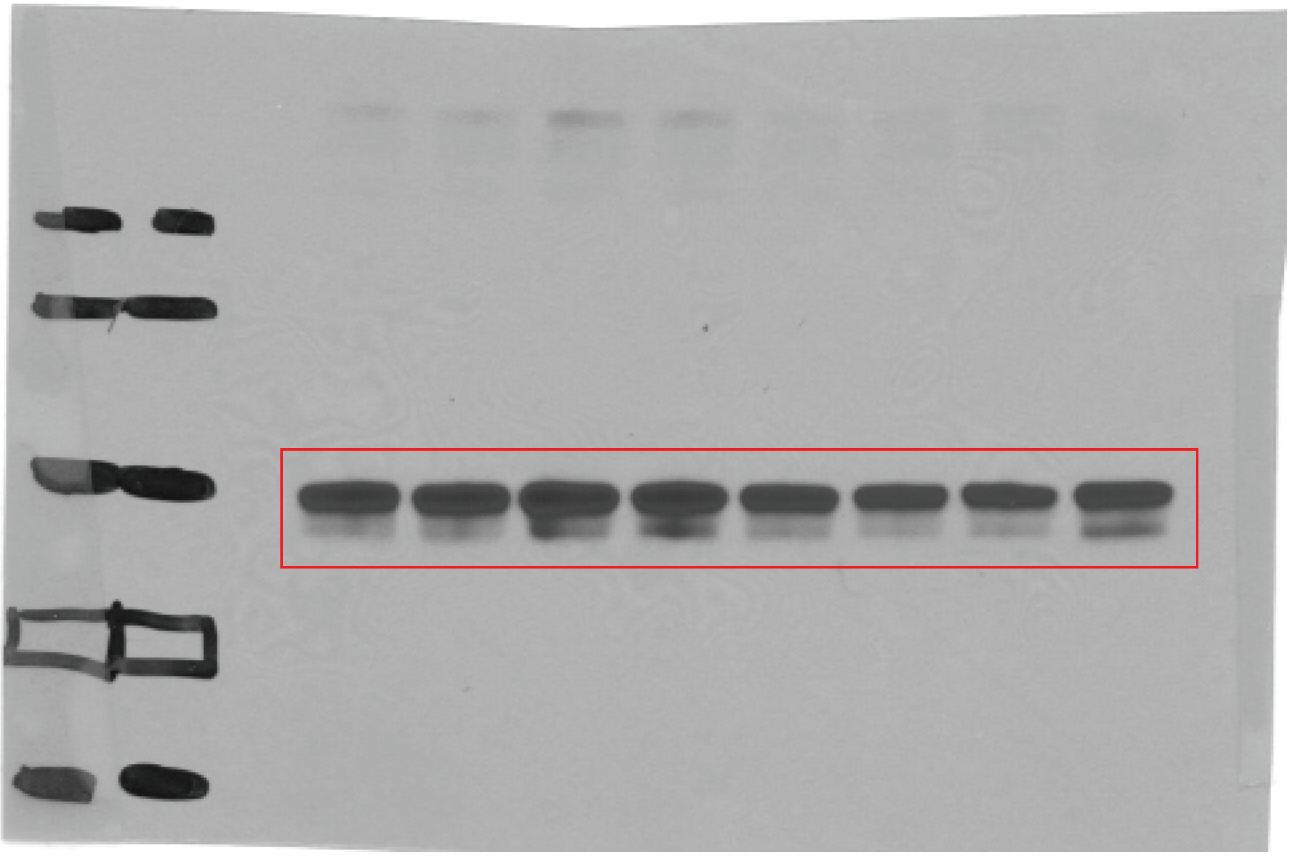

Figure 4a

Figure 4, part 2

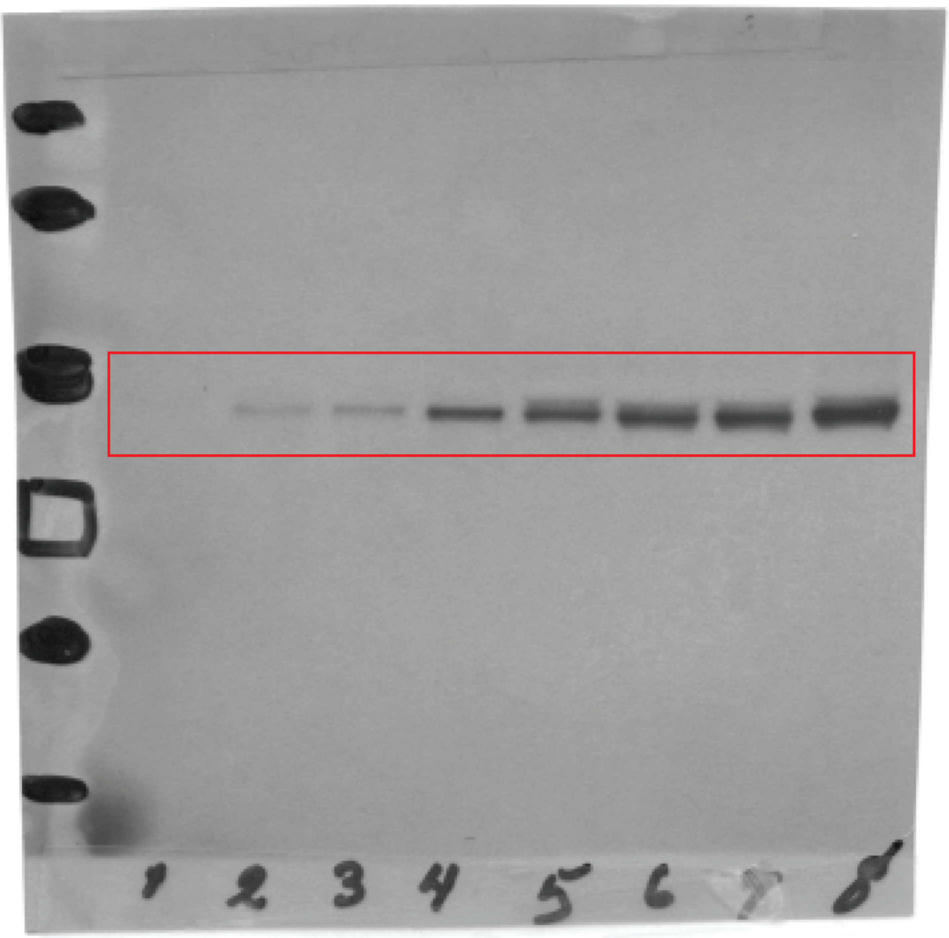

Figure 4a

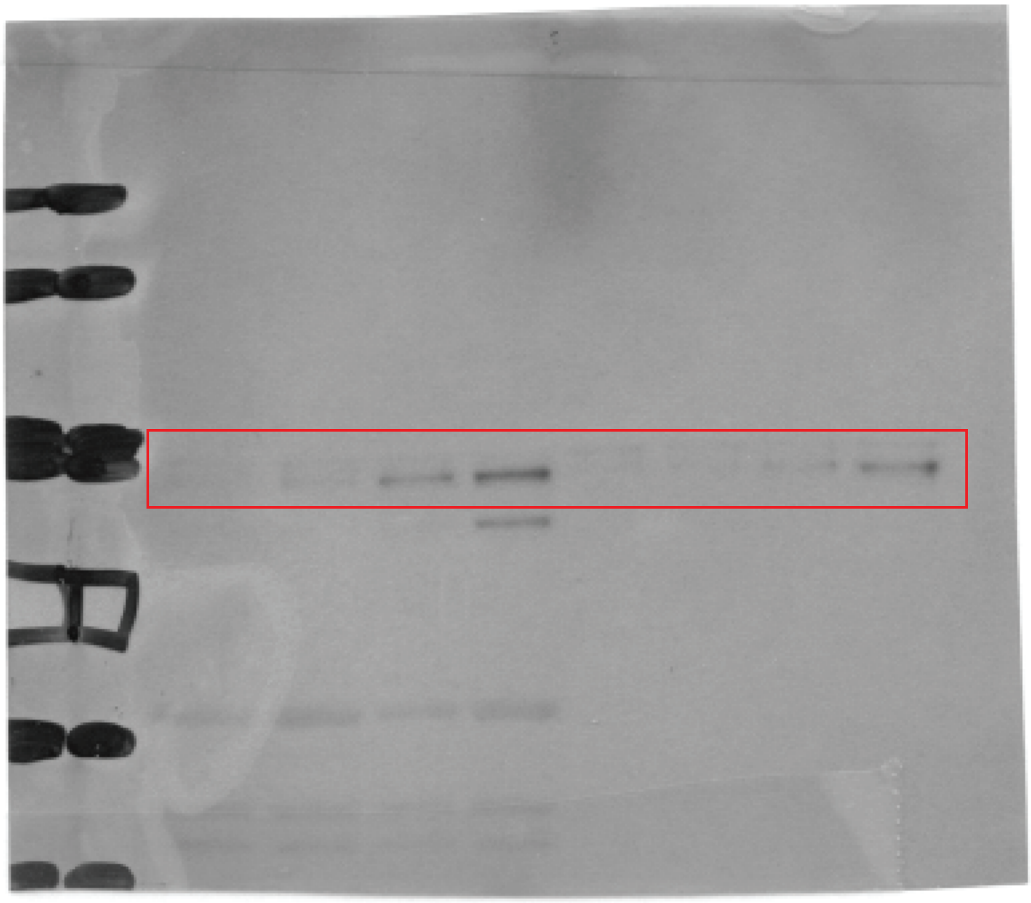

Figure 4a

Figure 4, part 3

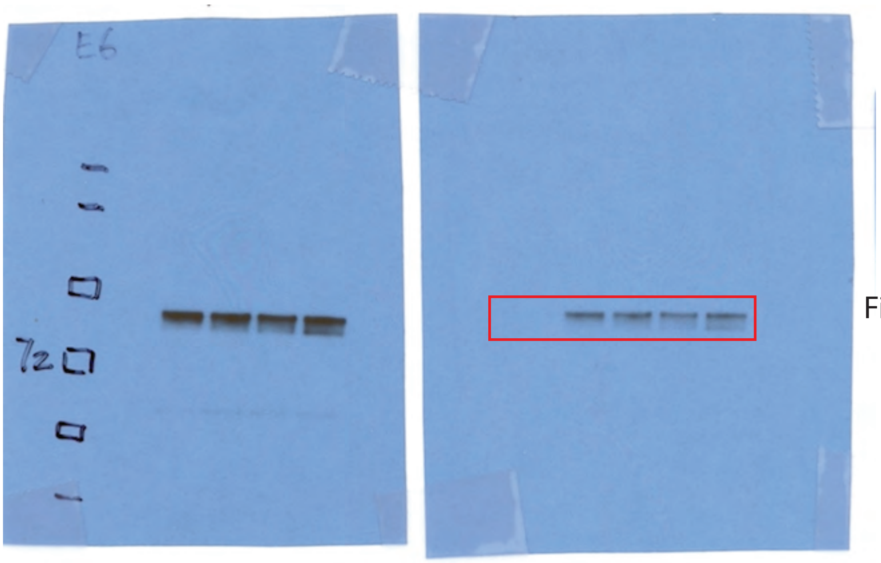

Figure 4b

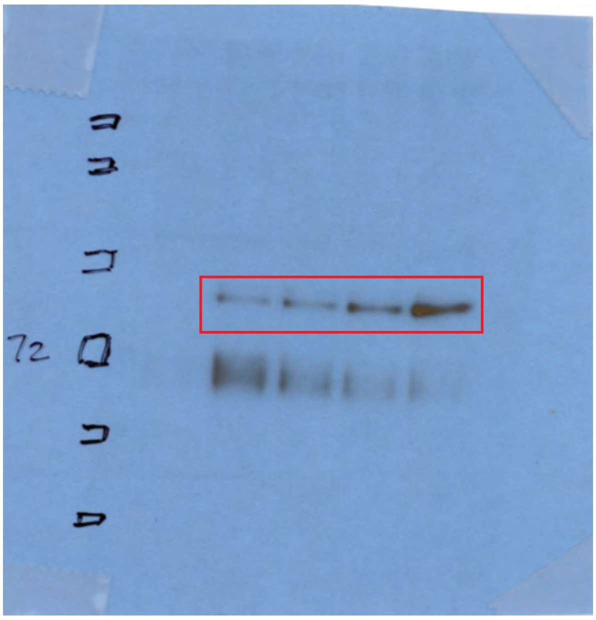

Figure 4b

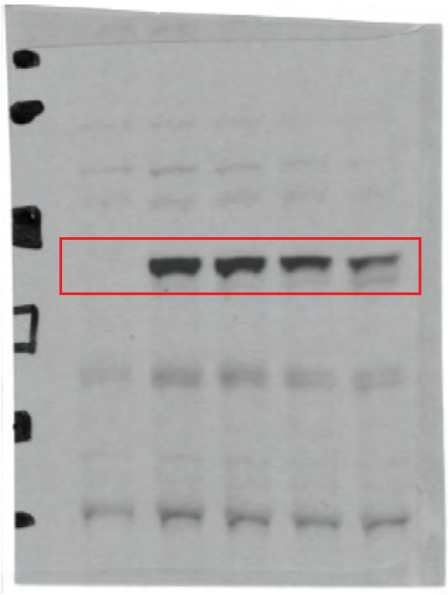

Figure 4b

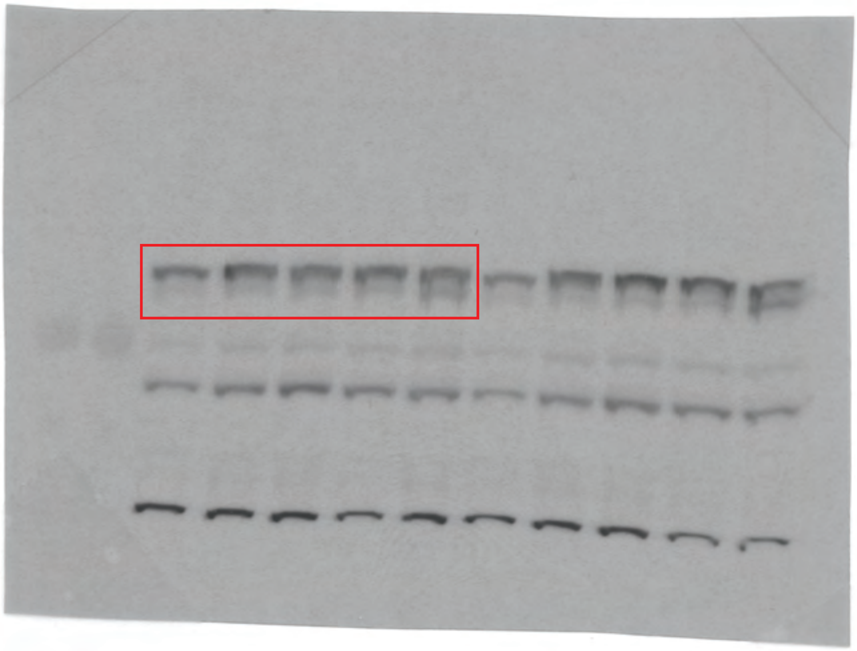

Figure 4b

Figure 4, part 4

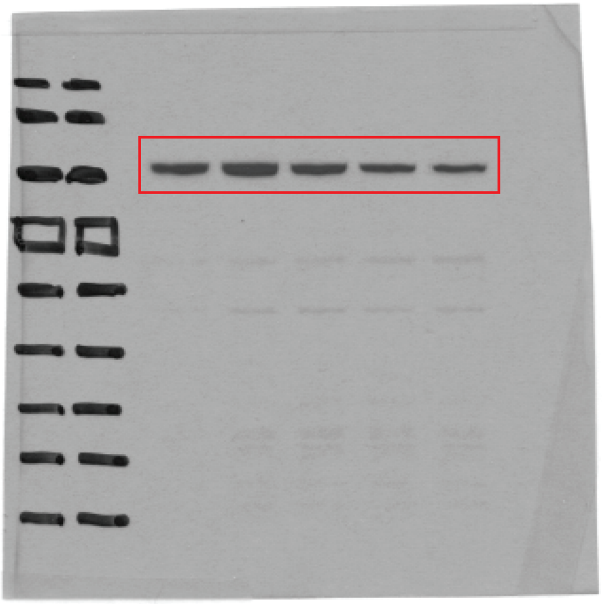

Figure 4b

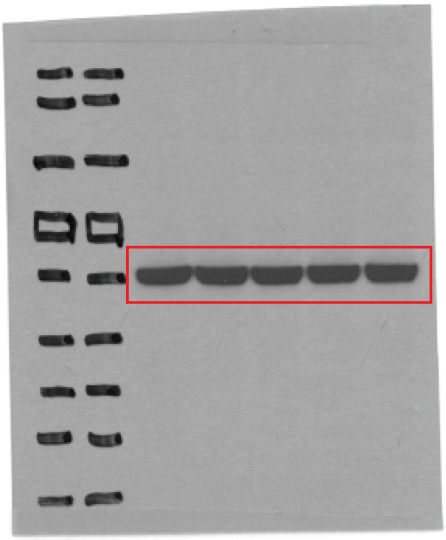

Figure 4b

Figure 4, part 5

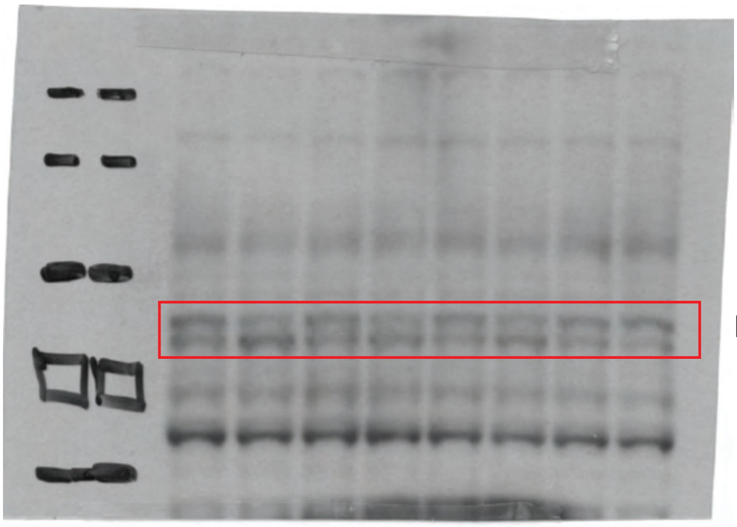

Figure 4c

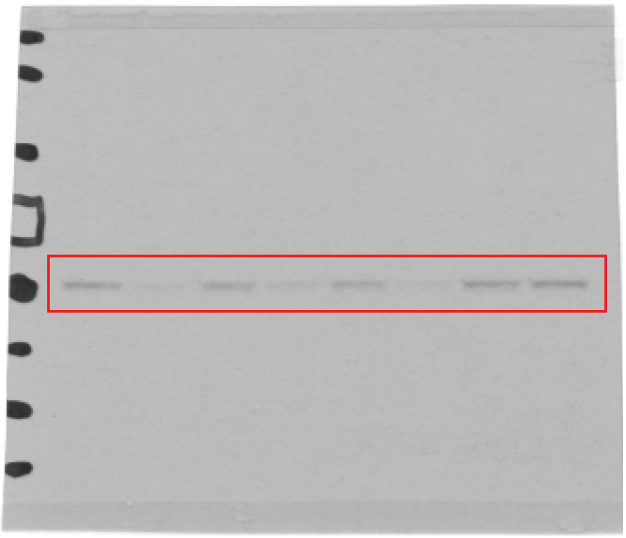

Figure 4c

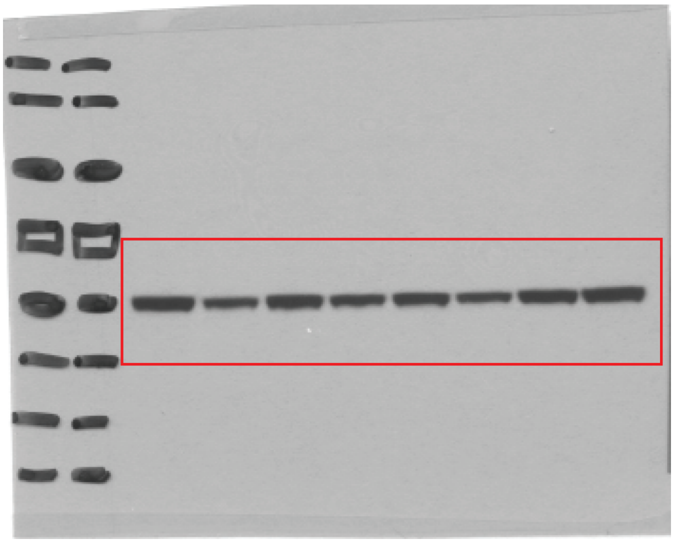

Figure 4c

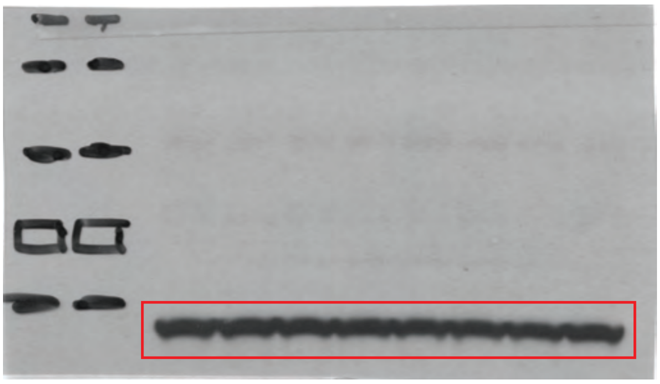

Figure 4c

Figure 4, part 6

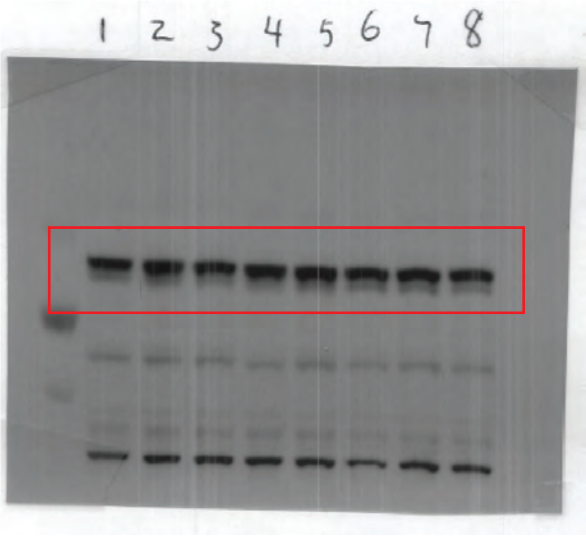

Figure 4c

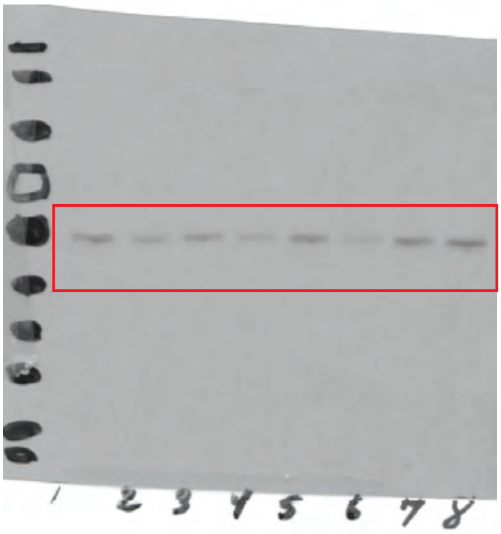

Figure 4c

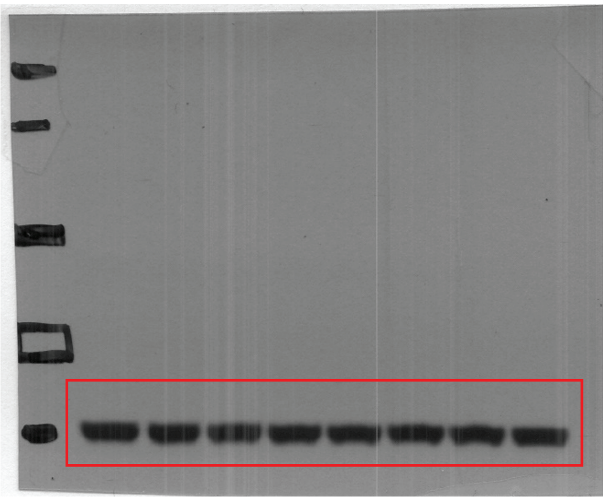

Figure 4c

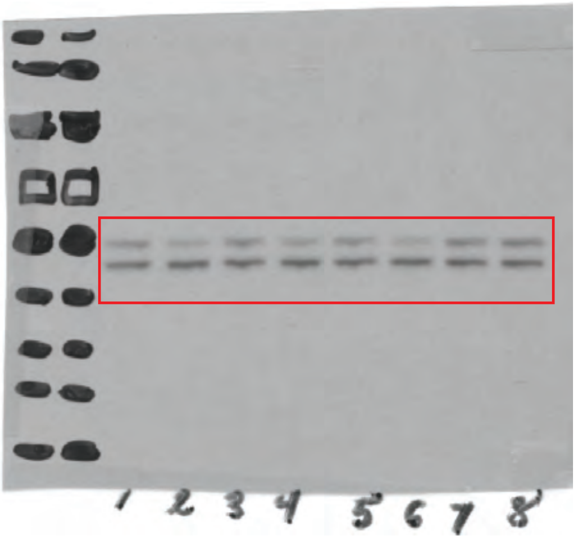

Figure 4c

Figure 5, part 1

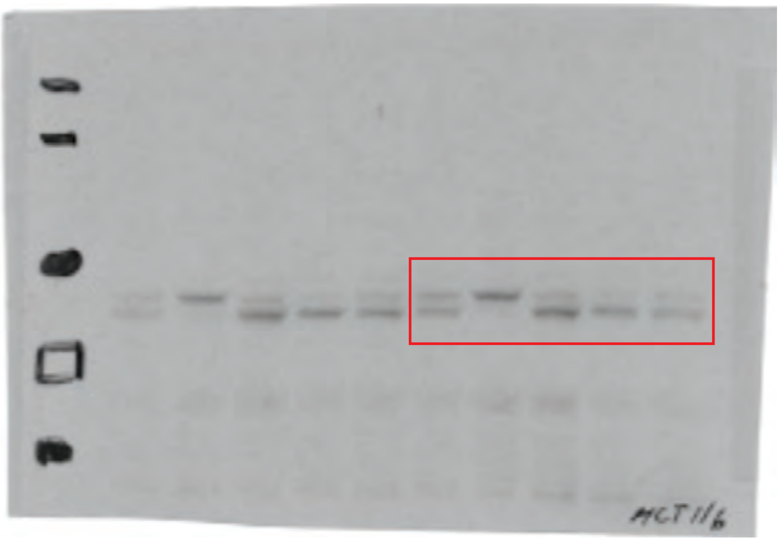

Figure 5a

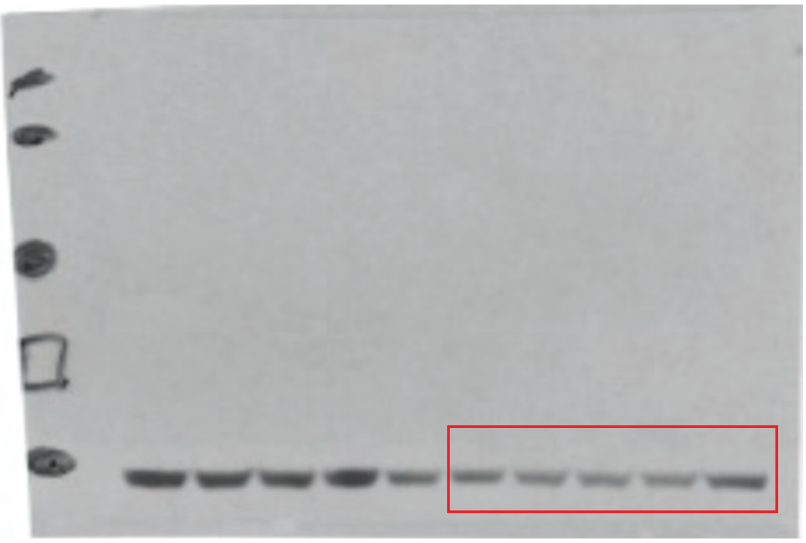

Figure 5a

Figure 5, part 2

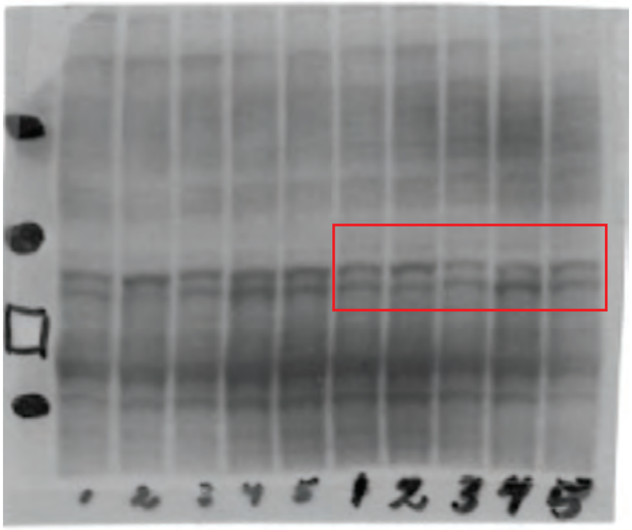

Figure 5b

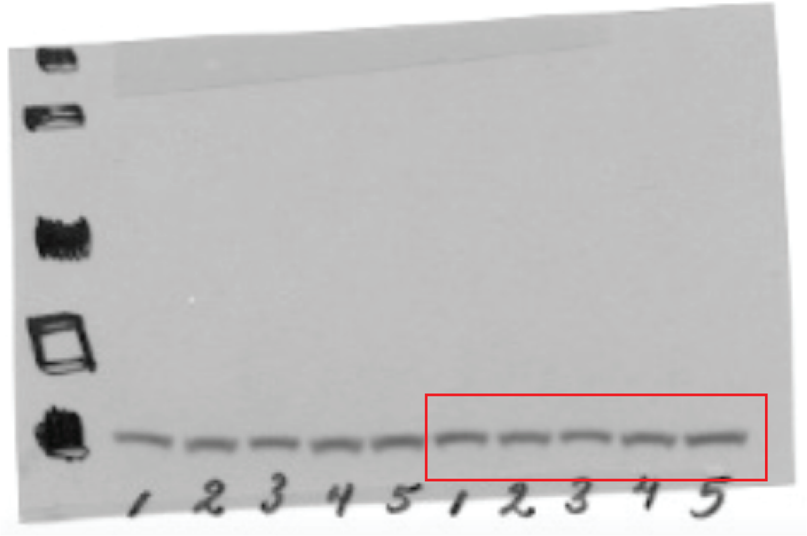

Figure 5b

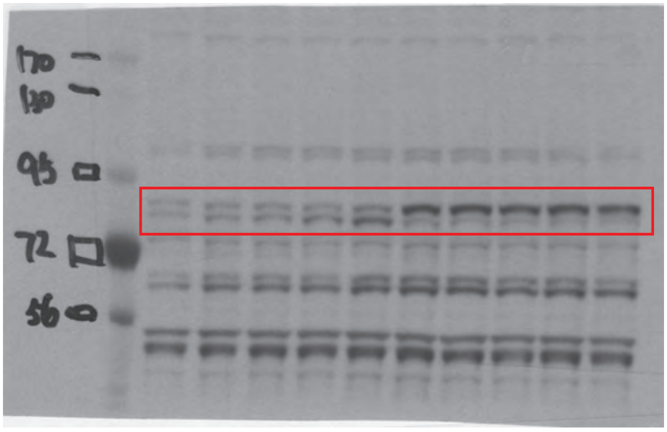

Figure 5c

Figure 5, part 3

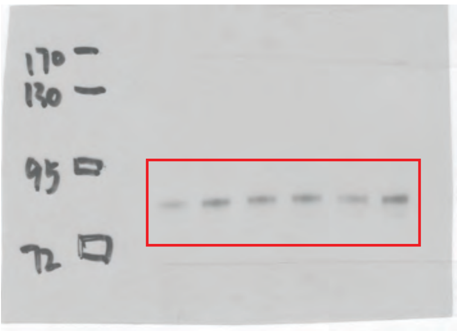

Figure 5e

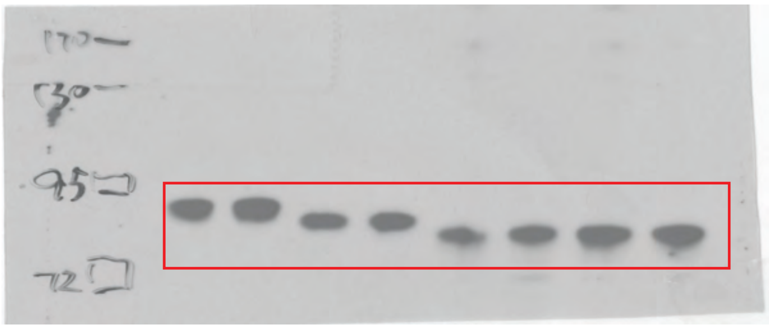

Figure 5f

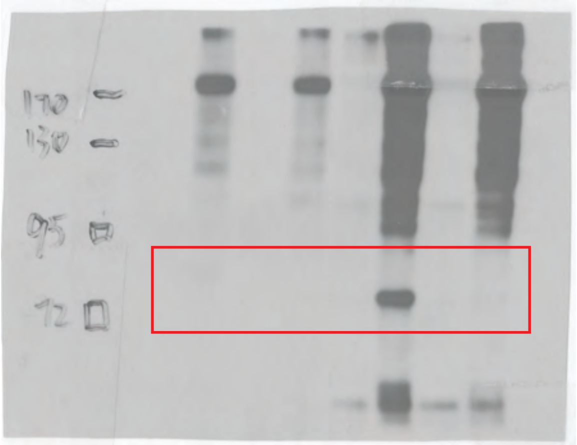

Figure 5f

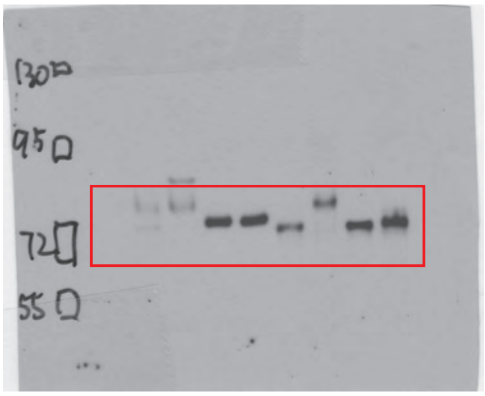

Figure 5f

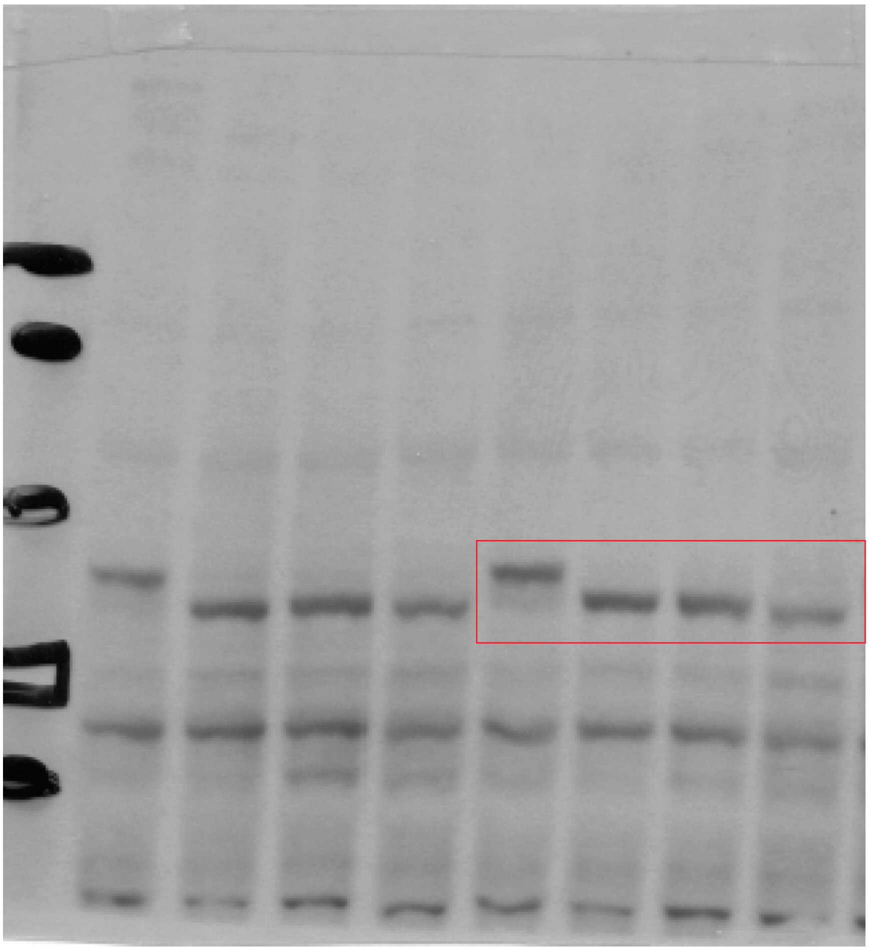

Figure 6a

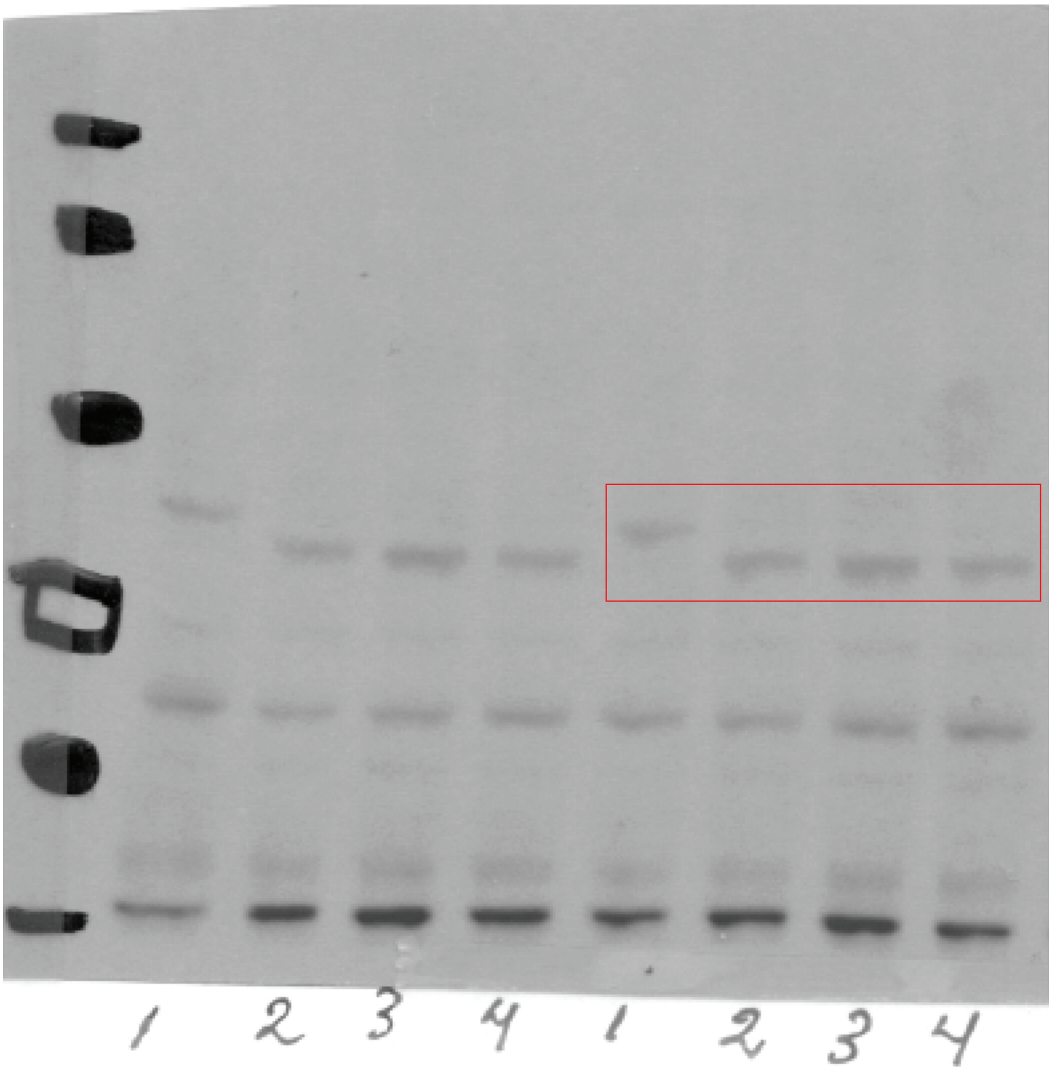

Figure 6b

Figure 6, part 2

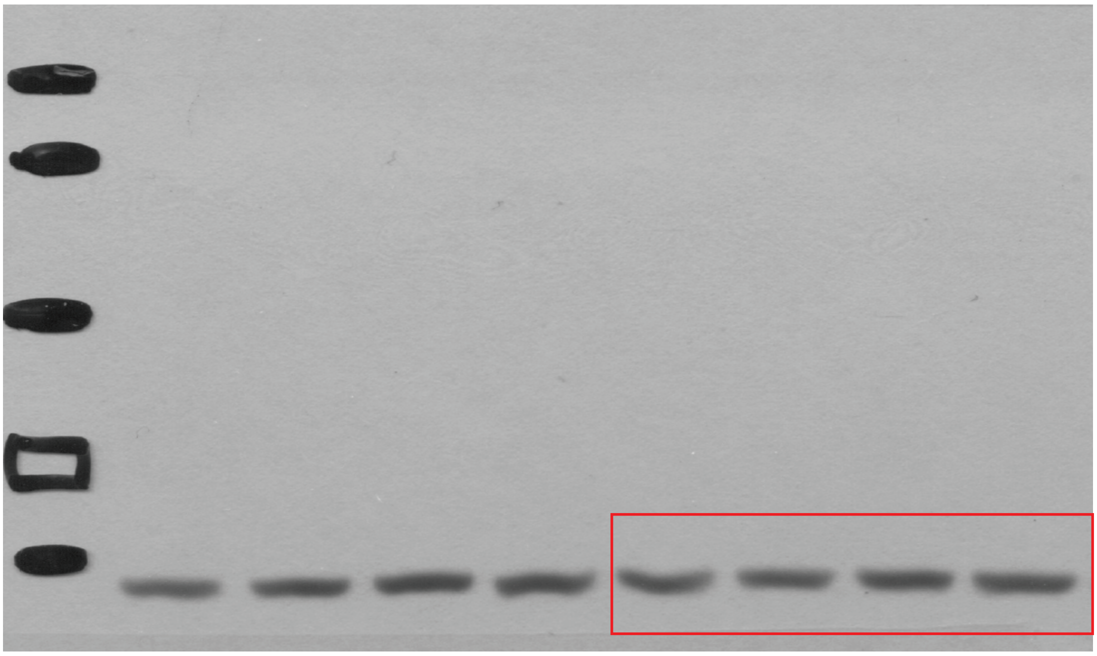

Figure 6a

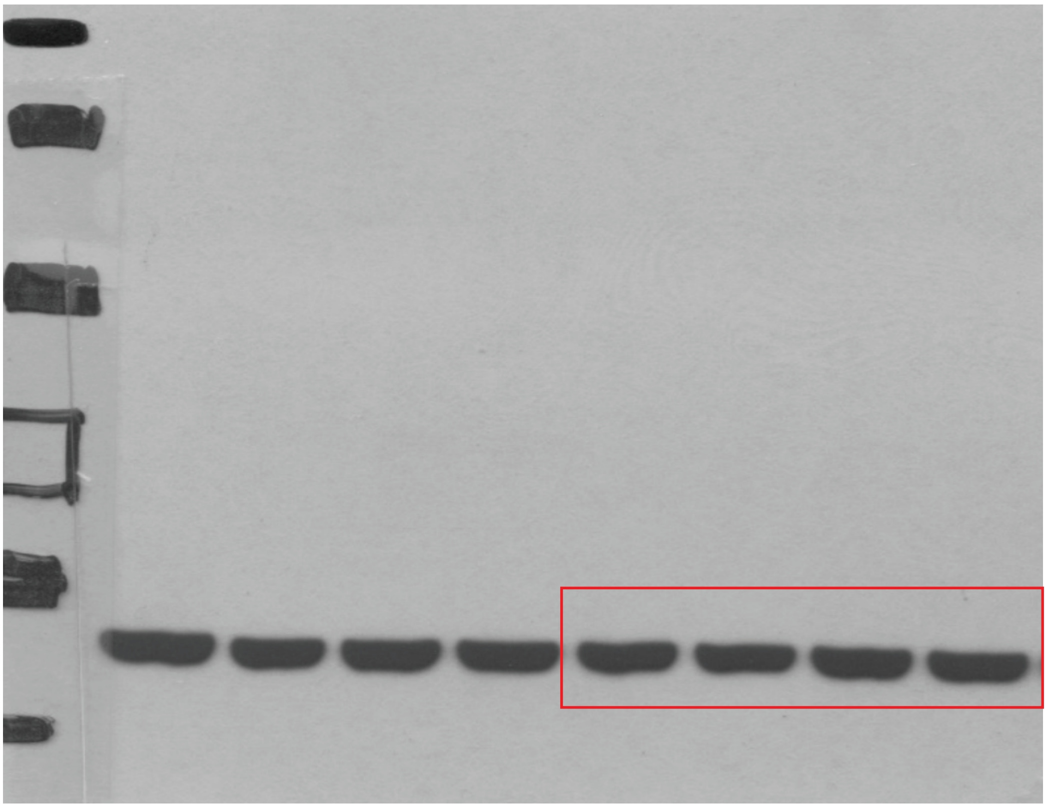

Figure 6b
